# Simple rules can guide whether land- or ocean-based conservation will best benefit marine ecosystems

**DOI:** 10.1371/journal.pbio.2001886

**Published:** 2017-09-06

**Authors:** Megan I. Saunders, Michael Bode, Scott Atkinson, Carissa J. Klein, Anna Metaxas, Jutta Beher, Maria Beger, Morena Mills, Sylvaine Giakoumi, Vivitskaia Tulloch, Hugh P. Possingham

**Affiliations:** 1 Centre for Biodiversity and Conservation Science, The University of Queensland, St. Lucia, Australia; 2 Australian Research Council (ARC) Centre of Excellence in Environmental Decisions, University of Queensland, St. Lucia, Australia; 3 School of Earth and Environmental Sciences, The University of Queensland, St. Lucia, Australia; 4 The Global Change Institute, The University of Queensland, St. Lucia, Australia; 5 School of Chemical Engineering, The University of Queensland, St. Lucia, Australia; 6 Australian Research Council Centre of Excellence for Coral Reef Studies, James Cook University, Townsville, Australia; 7 Department of Oceanography, Dalhousie University, Halifax, Nova Scotia, Canada; 8 School of Biology, University of Leeds, Leeds, United Kingdom; 9 Department of Life Sciences, Imperial College London, Silwood Park Campus, Buckhurst Road, Ascot, Berkshire, United Kingdom; 10 Université Côte d’Azur, CNRS, FRE 3729 ECOMERS, Parc Valrose, Nice, France; 11 The Nature Conservancy, Arlington, Virginia, United States of America; University College London, United Kingdom of Great Britain and Northern Ireland

## Abstract

Coastal marine ecosystems can be managed by actions undertaken both on the land and in the ocean. Quantifying and comparing the costs and benefits of actions in both realms is therefore necessary for efficient management. Here, we quantify the link between terrestrial sediment runoff and a downstream coastal marine ecosystem and contrast the cost-effectiveness of marine- and land-based conservation actions. We use a dynamic land- and sea-scape model to determine whether limited funds should be directed to 1 of 4 alternative conservation actions—protection on land, protection in the ocean, restoration on land, or restoration in the ocean—to maximise the extent of light-dependent marine benthic habitats across decadal timescales. We apply the model to a case study for a seagrass meadow in Australia. We find that marine restoration is the most cost-effective action over decadal timescales in this system, based on a conservative estimate of the rate at which seagrass can expand into a new habitat. The optimal decision will vary in different social–ecological contexts, but some basic information can guide optimal investments to counteract land- and ocean-based stressors: (1) marine restoration should be prioritised if the rates of marine ecosystem decline and expansion are similar and low; (2) marine protection should take precedence if the rate of marine ecosystem decline is high or if the adjacent catchment is relatively intact and has a low rate of vegetation decline; (3) land-based actions are optimal when the ratio of marine ecosystem expansion to decline is greater than 1:1.4, with terrestrial restoration typically the most cost-effective action; and (4) land protection should be prioritised if the catchment is relatively intact but the rate of vegetation decline is high. These rules of thumb illustrate how cost-effective conservation outcomes for connected land–ocean systems can proceed without complex modelling.

## Author summary

Many coastal marine ecosystems are threatened by anthropogenic activities, but often, the best way to restore and protect these important ecosystems is unclear. Conventional wisdom suggests that the 2 most effective conservation actions to benefit coastal marine ecosystems are implementation of marine protected areas or, alternatively, reduction of land-based threats. Active marine restoration is typically considered a low-priority option, in part due to high costs and low success rates. But does this conventional wisdom hold up to closer scrutiny? We developed a model to ask: should we restore or protect, on either the land or in the ocean, to maximise the extent of coastal marine ecosystems? We based the model on seagrass meadows and adjacent catchments in Queensland, Australia. Surprisingly, we found that direct, active marine restoration can be the most cost-effective approach to maximising extent of marine ecosystems over longer (decades-long) timescales. There is, however, substantial uncertainty in our understanding of the dynamics of complex linked land–sea ecosystems. Further, geomorphological and ecological conditions vary geographically. Therefore, we also used the model to investigate how uncertainty in key parameters affects decision-making outcomes. Our results can be used to guide investment into coastal marine conservation in the absence of complex, region-specific modelling.

## Introduction

Widespread degradation and loss of coastal marine ecosystems has occurred over the previous centuries and has accelerated in recent decades [1–5]. These changes compromise the delivery of important ecosystem services to human society [6]. Coastal marine ecosystems pose a particular challenge to environmental managers because they are exposed to threats occurring both in the ocean (e.g., overfishing, direct damage) and on land. The conversion of native terrestrial vegetation for agriculture, urbanization, and industry increases runoff [7], causing degradation and die-offs of coastal ecosystems such as coral reefs [8] and seagrass meadows [2]. These declines threaten the functional integrity of coastal and marine ecosystems and the services they provide, such as food supplies, coastal protection, and climate regulation [9–11]. Consequently, the conservation of coastal species and ecosystems requires a mixture of both marine and terrestrial conservation actions [12–16].

Conservation prioritisation of marine ecosystems and adjacent landscapes traditionally focuses on protecting intact habitats, in either marine and or terrestrial realms, from future degradation [e.g. 17, 18–20] [but see 21]. Ecological restoration is commonly considered a less preferred management strategy than protection [22], particularly in marine environments, where restoration costs are high and success rates are low [23]. However, restoration can deliver better ecological outcomes than protection, depending on existing land uses, conservation intervention costs, and ecosystem expansion rates [24]. Compared to other actions, restoration is rarely considered [25], and trade-offs between restoration and protection actions have never been evaluated across complex land–sea systems.

Comparing the costs of conservation actions, both on land and in the ocean, with the benefits accrued in the marine ecosystem (‘cost-effectiveness’) is at the forefront of conservation planning for land–sea ecosystems [e.g. 17]. Incorporating exchanges across the land–sea interface is challenging, requiring the integration of data and models across the terrestrial, freshwater, and marine realms [12–14, 20]. Recent advances have allowed the benefits of terrestrial actions on marine ecosystems to be estimated [17–20, 26–33], but in practice, land–sea conservation planning has rarely explicitly quantified how the management of terrestrial threats impacts marine ecosystems. For instance, recent implementations of the ‘Reef 2050 Long-term Sustainability Plan’ for the Great Barrier Reef and the ‘Chesapeake Bay Total Maximum Daily Load’ programs aim to minimise sediment, nutrient, and pollutant delivery to the ocean and assume that marine ecosystems will respond positively [34, 35] but do not predict the effect size of the marine ecosystem response. As a result, it is not clear that their terrestrial focus will outperform actions in the marine environment, which have the advantage of directly affecting the management goal. To compare and prioritise actions across the land–sea interface, we need to identify the links between (1) the amount of land-based actions required to reduce a threat on receiving marine environments and (2) the amount of change in the marine ecosystem triggered by such a reduction.

**Fig 1:**
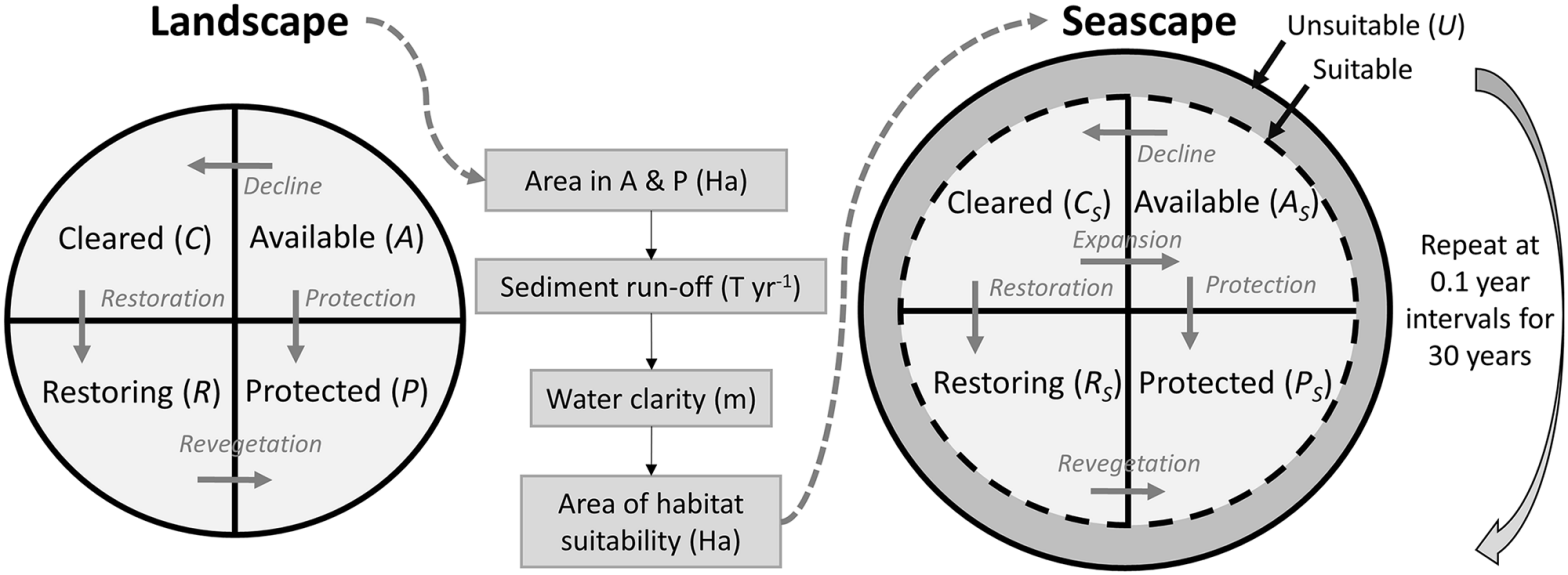
Conceptual diagram of the dynamic land- and sea-scape model used to identify how investment in conservation actions (restoration or protection) on land or in the ocean affects the extent of marine habitats. The Cleared (C), Available (A), Restoring (R), and Protected (P) categories on land and ocean indicate habitat area as a proportion of the land- and sea-scape, respectively, such that C + A + R + P = 1 and C_S_ + A_S_ + R_S_ + P_S_ = 1. The area of suitable habitat in the seascape changes in each time step as a function of the area of intact (Available or Protected) land in the landscape, which in turn modifies sediment loads delivered to the ocean.

We propose that integrated land–sea planning must compare the cost-effectiveness of 4 broad conservation actions: protect habitat on the land, protect habitat in the ocean, restore habitat on the land, and restore habitat in the ocean. Here, we develop a repeatable and transferable approach to determine which of those 4 actions maximises the extent of intact marine habitat for a given budget and project timeframe (Fig 1). The model extends an existing terrestrial model [24] across the land–sea interface. It is general in structure and could potentially apply to any marine system that is affected by sediment runoff and marine-based threats. In the original terrestrial model [24], the landscape is divided into 4 states describing the condition of the native vegetation—intact and unprotected, intact and protected, cleared, or restoring. The act of restoring or protecting habitat moves it between these different states. In our expanded model, we consider the state of habitat in both a landscape and adjacent seascape, which are connected by sediment runoff from the land into the ocean. Cleared terrestrial habitat increases sediment loads, which reduces water clarity in the adjacent ocean. The resulting decrease in light reaching the seafloor reduces the amount of habitat suitable for light-dependent species [13]. We focus on suspended sediments because they are a key driver of marine ecosystem condition in many inshore areas [2, 8, 36] but acknowledge the importance of the other components of runoff more broadly, including toxicant and nutrient loads. Importantly, our model assumes that the marine ecosystem is sensitive to both sediment runoff [37] and marine-based threats and that there is habitat in appropriate condition for marine restoration; if these conditions are not met, then approaches targeted towards either land- or ocean-based threats would be required. The model is spatially implicit, i.e., it is parameterised by spatial data (see Materials and methods, S1 Text, S1 Table).

**Fig 2:**
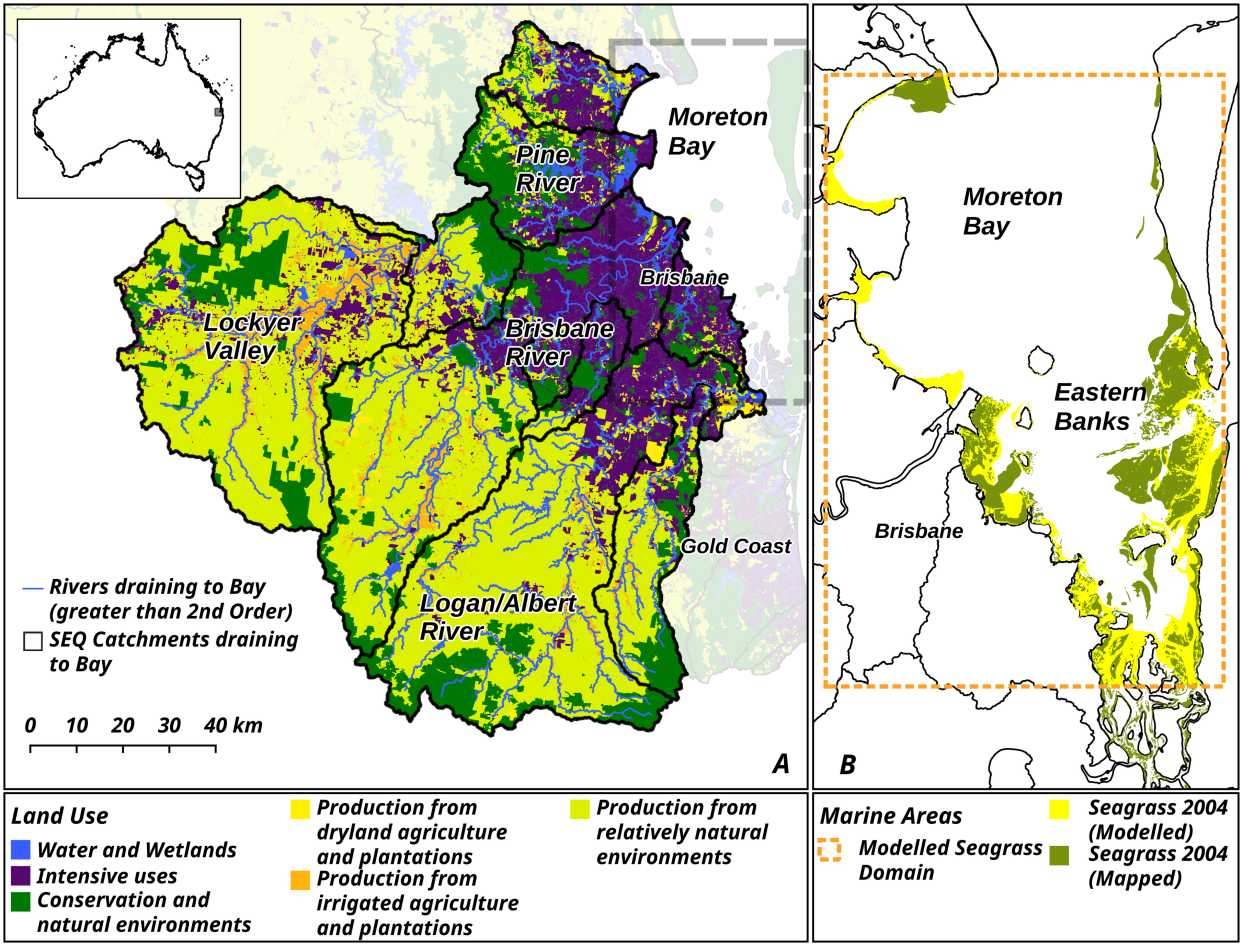
Study site in Queensland, Australia used to quantify how investment in conservation actions (restoration or protection) on land or in the ocean affects the extent of marine habitats (seagrass). See Materials and methods for data sources.

We apply this model to a case study of seagrass meadows in Moreton Bay and riparian areas in adjacent catchments in Queensland, Australia (Fig 2). Seagrass meadows are an excellent test system because they provide a suite of ecosystem services and are strongly influenced by both land-based processes and direct local impacts in the ocean [2, 38–40]. The catchments draining into Moreton Bay are heavily modified, with only 20% intact remnant vegetation. Historical and ongoing land-clearing has significantly increased soil erosion, primarily through the process of gully erosion [41–42]. Suspended sediment delivery has negatively impacted marine ecosystems in the region [43–45]. The site is therefore representative of the wider global challenges posed to marine ecosystems by increased sediment runoff [14, 15, 39].

We ultimately aim to identify key factors that determine which broad conservation action is most effective under different circumstances. Therefore, we use the model output and sensitivity analyses to answer 2 questions. One, which of the 4 conservation actions maximises the extent of intact seagrass after 30 years? And two, under which conditions would our decision-making vary? Using the results from this and other studies [24, 37], we propose simple ‘rules of thumb’ that can help decision makers identify whether restoration or protection, either on the land or in the ocean, will be the most cost-effective approach to improving the state of marine ecosystems. These rules of thumb are likely specific to our study system but may be used as guidelines (or, alternatively, viewed as hypotheses) to inform decision-making in other regions until models are parametrised for those sites.

## Results

**Fig 3:**
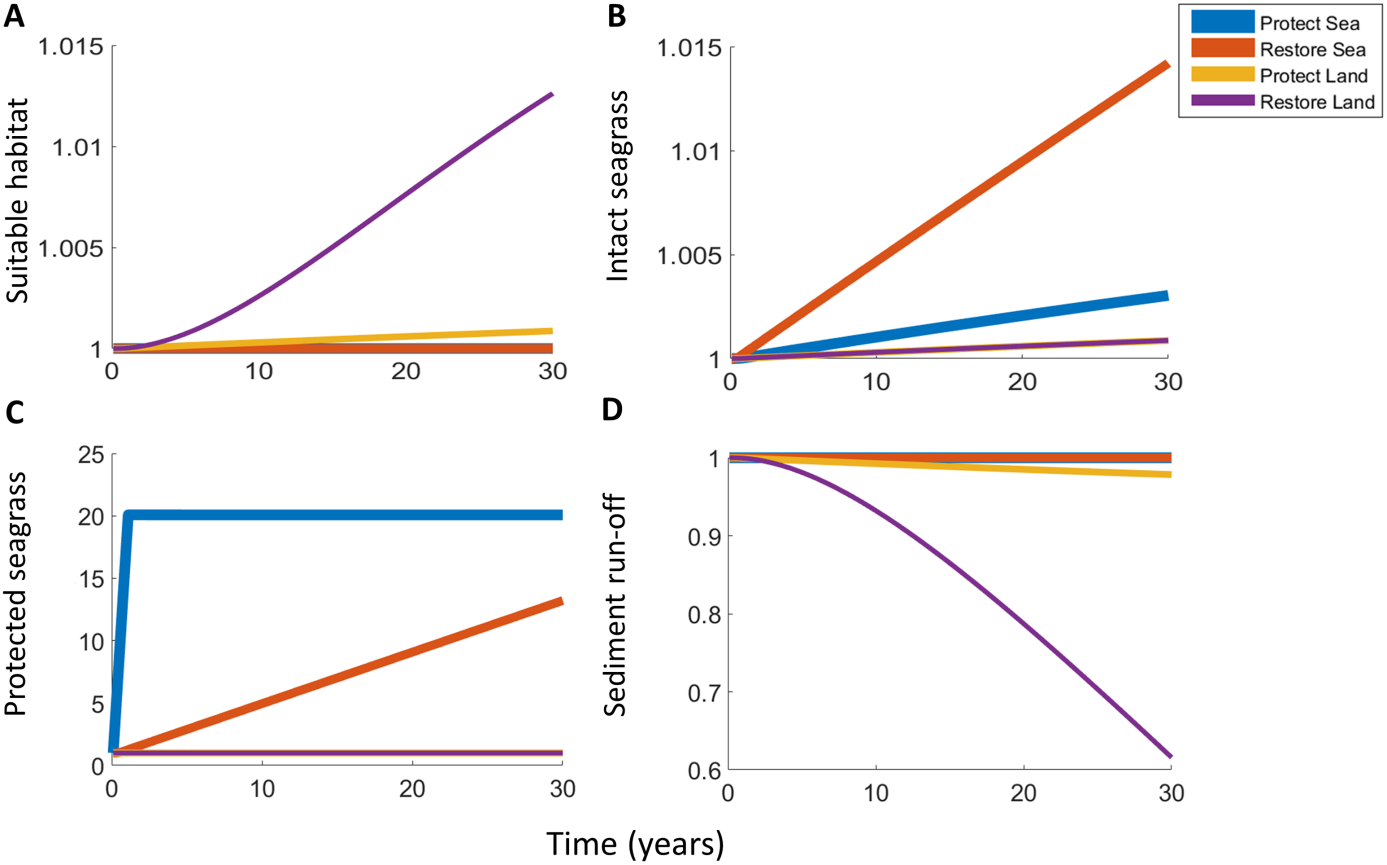
Effects of $50 million per year investment in each of 4 conservation actions, restoration or protection on land or in the ocean, on marine ecosystems. (A) area of suitable (but not necessarily occupied) marine habitat; (B) area of intact marine habitat; (C) area of protected intact marine habitat; (D) annual sediment load. Y-axis is proportional to values which would have been achieved with no investment. Lines have varying thicknesses so that overlapping lines are visible.

Using our dynamic model of seagrass meadows and riparian areas in adjacent catchments in Southeast Queensland, Australia, we investigated the effect of investment of $50 million (all costs are in 2015 USD unless otherwise stated) per year over 30 years into each of 4 separate conservation actions. The model was used to quantify the area of intact seagrass habitat resulting from marine restoration, marine protection, terrestrial restoration or terrestrial protection (see below, Materials and methods and S1 Text, for detailed descriptions). We found that if the objective is to increase the amount of habitat suitable for (but not necessarily occupied by) light-sensitive species (in this case, seagrass), then restoration of riparian areas on land is the most cost-effective strategy (Fig 3A). However, this will not necessarily maximise the area of occupied (‘intact’) marine habitat immediately, as that depends on how fast the marine ecosystem can recover and expand into habitat which was previously unsuitable due to low light availability; there is substantial uncertainty in this parameter (S2 Table). Controversially, we find that the most cost-effective way to maximise the extent of intact marine habitat over decadal timescales is to directly restore the marine ecosystem, despite the higher cost [23] (Fig 3B). Obviously, this conclusion depends on the availability of suitable, unoccupied habitat. If all marine habitat is unsuitable for marine restoration due to low water clarity, then revegetation of riparian vegetation to minimise sedimentation stress is required [46]. Below, we discuss the costs and benefits of each specific conservation action in turn for our study system and then examine how these decisions may vary for other systems.

Marine restoration was defined as planting seagrass transplants into habitat that is suitable for, but not presently occupied by, seagrass and was the most cost-effective action for achieving the highest coverage of seagrass habitat after 30 years (Fig 3B). Our modelling assumes that: (1) it takes 3 years for seagrass transplants to grow, fill in meadow gaps, and become a ‘healthy’, self-sustaining meadow [47]; (2) it costs $418,000 per ha [23] to source, transplant, and monitor the seagrass; (3) restoration has a high failure rate (62%) [23]; and (4) a maximum of 0.1% of the existing meadow can be in a restoring state in any year. Surprisingly, despite an expected cost of over $1 million per ha for successful restoration, restoring seagrass is a better strategy to maximise seagrass coverage than marine protection or land-based actions. Larger areas of intact seagrass are achieved if the area of seagrass that is in a restoring state in any year is less conservative (e.g., 1% [S1 Fig]). Our results imply that, given sufficient funding, effort, and suitable habitat, large-scale marine restoration projects could achieve significant gains in ecosystem extent, as recently assessed [46].

Marine protection was defined as the installation of environmentally friendly moorings, which avoid seafloor damage caused by traditional moorings and minimise the effects of dragging anchor chains [48]. In other regions, where trawling or dredging are the main threats to seagrass, the implementation of Marine Protected Areas (MPAs), which minimise seafloor damage by excluding destructive activities, may be a more appropriate conservation action [49]. Our study area is a Marine Park where seafloor destructive fishing techniques are forbidden and environmentally friendly moorings are the approach currently used to increase seagrass protection [50]. Our model predicts that marine protection yields the fastest initial increase and greatest total area of protected seagrass habitat (Fig 3C), because it is relatively cheap compared to the other actions. Marine protection increased the overall area of seagrass through time by a small amount (Fig 3B), because seagrass habitat decline rates are proportional to the amount of unprotected habitat. At $131,000 per ha, the 8.8% of seagrass habitat that is suitable for protection in the study region (S1 Text) can be protected in the first year so that, over decadal scales, the impact of marine protection on seagrass habitat area is limited.

Land restoration was defined as using revegetation and other actions in the riparian zone to reduce erosion in riverine locations where native vegetation had been previously cleared, at a cost of $17,310 per ha and with a probability of success of 50% (personal communication, J. O’Mara, SEQ Catchments). The resulting reduction in runoff (Fig 3D) increases the area of suitable marine habitat (Fig 3A), as the increased water clarity improves light availability on the seafloor. Our model advances and operationalises our understanding of the impacts of sediment input on light-dependent benthic marine species by factoring in an ‘action–response curve’ [51] describing the relationship between sediment loads and illuminated seafloor area that is suitable for light-dependent species (S1 Text). This relationship was generated using modelled daily sediment loads, monthly observed water clarity, and a species distribution model of seagrass habitat (S1 Text) [52] and is applicable in geomorphic and ecological contexts where sediment runoff impacts marine ecosystems [37]. Our results show that land restoration only offers small increases in seagrass coverage (Fig 3B) because there is a substantial 10-year time lag between restoration actions and the mitigation of sediment erosion and because we estimate that seagrass colonises newly available areas slowly (1.13% per year, [53]) (see S1 Text). Varying this parameter changes the results substantially, which we explore further below.

Land protection was defined as purchasing privately held land containing intact native vegetation and designating it as a nature reserve, at a relatively low cost of $3,530 per ha (S1 Text). Land protection only provides second-order benefits to marine habitat (Fig 3D): It reduces terrestrial habitat decline rates, leading to relatively less erosion and less sediment within the rivers. It therefore had relatively little impact on any metric of seagrass habitat (Fig 3A, 3B and 3C). Land protection therefore offers little benefit to catchments that are already highly degraded and where riparian habitat decline rates are low, such as in our case study.

While the model presented here can in theory be applied to any sensitive marine ecosystem affected by both land- and ocean-based threats, it is not straightforward to source the data needed for accurate parameterisation. We therefore varied key model parameters to identify contexts where the optimal conservation strategy may differ from our results, including rates of marine ecosystem decline and expansion, rates of terrestrial ecosystem decline, and the magnitude of previous land clearing. For instance, we can find the optimal investment strategy for landscapes with extensive historic and ongoing land clearing, such as parts of Malaysia and Indonesia [54], or high magnitudes of degradation but lower rates of ongoing land clearing, such as our study system [41] and Mediterranean countries including Albania, Algeria, and Bosnia [55]. Similarly, we can identify optimal approaches to marine ecosystems with different rates of habitat decline and expansion. For instance, kelp beds can undergo rapid declines yet can also recover rapidly when conditions are suitable [56]. In contrast, *Posidonia oceanica* seagrass meadows in some Mediterranean regions, such as Corsica, are declining slowly [57] but also have slow expansion rates [58].

**Fig 4:**
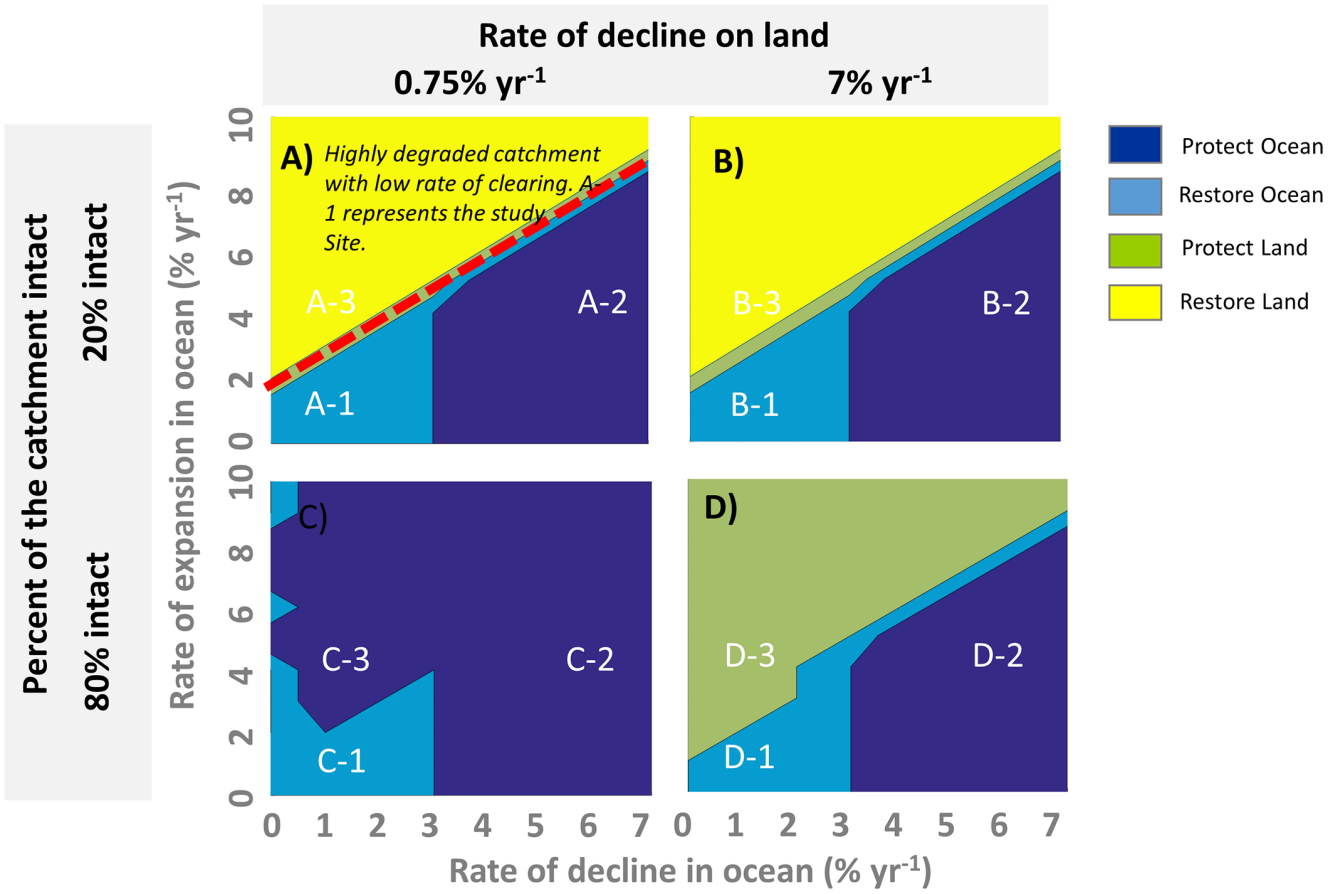
Impact of $50 million per year investment in land- or ocean-based conservation actions on marine ecosystems affected by land- and ocean- based impacts after 30 years. Panels A–D give results for 4 different ecological contexts for the catchment and marine ecosystems. Two parameters representing the landscape are varied: percentage of the catchment that is intact (e.g., % remnant vegetation) and the background rate of decline of habitats on land (percent per year). X- and Y-axes indicate the rate of expansion (recovery) and decline (percent per year) of the marine ecosystem. In A, ‘A-1’ is thought to best represent the study system (marine restoration is predicted to be the most cost-effective action), whereas ‘A-2’ and ‘A-3’ highlight contexts where marine protection and terrestrial restoration would be the most cost-effective actions, respectively. Letter–number pairs are used to guide Fig 5.

We discovered that the relative rates of decline and expansion in the marine ecosystem, as well as the rate and magnitude of degradation on land, are key factors in our decision-making (See Fig 4, S2, S3 and S4 Figs). When marine habitat can recover more quickly than the rate of marine habitat decline, then land-based conservation can yield optimal results. Specifically, if the ratio of marine habitat expansion to decline rates is greater than approximately 1:1.4 (ratio of x- and y-axis values indicated by red dashed line in Fig 4A), then actions on land typically deliver the greatest cost-effectiveness (Fig 4, A-3, B-3, D-3, but see C-3 below). In that case, land restoration is the best option (Fig 4A and 4B, A-3, B-3), unless the catchment is relatively intact and the rate of land decline is high, in which case land protection is most cost-effective (Fig 4D, D-3). If the catchment is relatively intact with a low rate of loss, then marine protection is optimal (Fig 4C, C-3). Conversely, if marine habitat decline rates are greater than expansion rates, we should act in the ocean, essentially regardless of what occurs on land. Specifically, if the ratio between expansion and decline rates for the marine ecosystem is less than approximately 1:1.4 (Fig 4, A-1, A-2, B-1, B-2, C-1, C-2, D-1, D-2), then we should act in the ocean. If the rates of marine ecosystem decline and expansion are similar and relatively low (less than 1% per year), then restoration in the ocean is the most cost-effective strategy (Fig 4, A-1, B-1, C-1, D-1). Marine protection is the most cost-effective action for our system when rates of seagrass decline outside MPAs are high (Fig 4, A-2, B-2, C-2, D-2).

**Fig 5:**
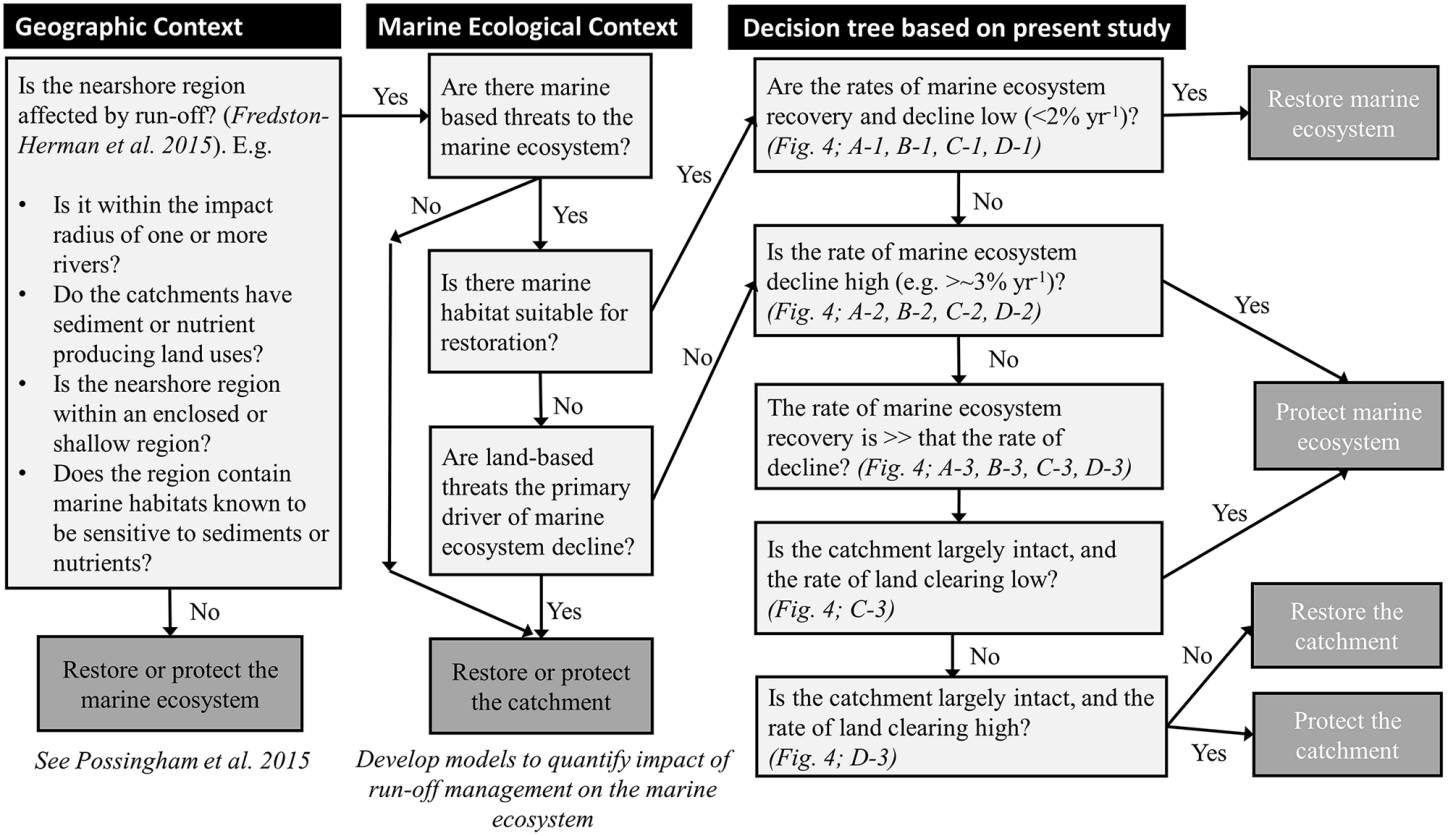
Flow chart of decision-making process for whether to take actions on land or in the ocean to best benefit marine ecosystems. Guidelines are based on [24, 37] and the modelling results in Fig 4 obtained using a dynamic landscape model.

The findings from our analyses are factored into a generic decision-making protocol for conservation investment in marine ecosystems influenced by both land- and ocean-based threats (Fig 5). A number of conditions must be met for land-based stressors to critically impact marine ecosystems [37]. In addition to the assumptions outlined previously, the nearshore marine region must be within the impact radius of 1 or more rivers and be in an enclosed or shallow region, and land uses within the catchments must have increased the erosion of sediments or nutrients on a large scale [37] (Fig 5). If these criteria are met, then the results from our model can be used as a first step to guide decision-making, without the need for complex modelling.

## Discussion

The conservation dynamics of coastal ecosystems are driven by a combination of terrestrial and marine drivers. Efficient conservation investment will require decision-support tools that are repeatable, transparent, and that quantitatively describe the connections between the land- and sea-scape. The optimisation model we describe here provides a robust and extendable method to support these decisions.

Our study drew an unexpected conclusion: Despite high costs and low success rates, direct restoration of marine ecosystems may be the most cost-effective method to maximise marine habitat extent over decadal timescales. If marine restoration is not likely to succeed due to poor water quality, lack of suitable substrate, or other factors, then this conclusion will clearly not hold true. Nonetheless, we propose that a paradigm shift is occurring, whereby restoration is being recognised in particular contexts as an important option for the recovery of biodiversity [24, 59]. This is supported by recent findings that marine restoration is more likely to succeed if it is conducted on larger spatial scales [46] and if care is taken to select appropriate sites and techniques [23].

Our study highlights several factors that are essential elements in determining the most cost-effective management action but which are not often present in decision-support tools. These include the effects of time lags and the complex, nonlinear relationship between activities on the land and benefits in the sea. Land-based impacts are key drivers of seagrass extent and condition, suggesting that terrestrial protection should be a high-priority conservation action [40]. However, from a management perspective, it is essential to compare the outcomes of actions against a specific objective and timeframe and to factor in economic constraints. Our findings align broadly with those of Klein et al. [60], who report that the cost-effectiveness of marine conservation was almost always higher than that of terrestrial conservation within any ecoregion in the Coral Triangle. Our findings differ from those of Gilby et al. [21], whose findings support the more commonly held view that the most effective actions to benefit inshore coral reefs in Moreton Bay, Australia, would be expansion of the marine reserve network and reductions in sediment inputs from land, without considering variation in the costs of management actions or time lags. Other studies have focused on quantifying the effects of land-based impacts or protection on marine ecosystems but have not compared the results to those obtained from protection from ocean-based threats or have not quantified the effects of restoration in either marine or terrestrial realms [18, 19, 31].

In reality, a combination of approaches—on land and in the ocean—will be required to achieve ecological improvements in many marine regions. For instance, land-based actions would be required first if there was no suitable marine habitat available for restoration (e.g., Fig 5). For our study system, all seagrass that is suitable for protection using environmentally friendly moorings could be achieved using the budget in the first year. Similarly, the budget for marine restoration was not completely allocated in a given year when we assume that only small areas of marine restoration can be achieved at one time. This means that marine protection or restoration could be implemented first and that the budget could be used for other strategies concurrently or in later years. In practice, budget and regulatory agencies often do not span the land–sea interface, which means land- and ocean-based actions will likely proceed independently of one another.

Our model predicts the area of seagrass habitat change resulting from management actions on either side of the land–sea interface. The modelling framework provides a substantial advance in our ability to quantify the costs and benefits associated with conservation actions on land and in the ocean. In the present study, our objective was to maximise the extent of seagrass, but, there are multiple benefits from catchment restoration that are unrelated to marine ecosystems, such as enhanced freshwater biodiversity, reduced drinking water treatment costs, and increased public amenity. If we instead aim to maximise the delivery of ecosystem services provided by both seagrass and riparian habitats, which are worth $26,226 per ha per year and $27,021 per ha per year in 2007 USD, respectively, based on [61], then the optimal decision would always be to restore the catchment (S1 Text, S5 Fig). Although we used habitat area as a proxy for the delivery of ecosystem functions and services by a habitat, metrics of habitat condition (e.g., cover, biomass, species composition) could be explored in the future. The ultimate aim of this approach is to identify the most cost-effective ways of maximising ecosystem functions and services (e.g., food or habitat for other species, water filtration, and wave attenuation).

There are a number of uncertainties and assumptions that affect model outputs and interpretation. First, we quantify the seagrass decline rate from satellite imagery in a clear water region [62], which is likely less impacted than nearshore areas and therefore may underrepresent the decline rate. Second, the rates at which marine organisms can colonise new areas vary among species and regions (S2 Table), are scale-dependent, and the factors influencing expansion rates are not well understood. Low seagrass expansion rates may indicate bistability of seagrass and base substratum systems, where feedback mechanisms hinder the re-establishment of vegetation [63]. High seagrass expansion rates following improvement of environmental conditions can occur in some contexts [64], typically when seagrass seed banks are present [65]. The influence of rates of seagrass decline and expansion are explored in the sensitivity analyses. Predicting the area that is suitable for restoration in marine habitats requires a habitat distribution model, the results of which contain several uncertainties, including whether all relevant environmental variables have been included in the model, whether the species is in equilibrium with environmental variables, and which method is used to select the threshold value delineating species presence and absence [52]. The effectiveness of catchment restoration is also uncertain; erosion may in fact temporarily increase following riparian restoration before eventually diminishing [66], although our uncertainty in the parameter representing time lags in restoration is not likely to impact whether we should be acting on land or in the ocean (S2 Fig). There is large variability in the costs and success rates of restoration [23], yet we do not find these to be the most important factors affecting whether actions should occur on land or in the ocean (S3 Fig). Furthermore, the costs and success rates of conservation actions will vary across the land- and sea-scape; when costs vary spatially, managers can target lower-cost areas preferentially, which reduces the average cost of management activities. Lastly, our analysis does not factor in the impact of nutrient runoff, pesticides and herbicides, climatic variability, extreme events, or climate change, the impacts of which are extremely challenging to predict [67] and therefore beyond the scope of our work but which are important areas of future research.

Further uncertainty lies in the impacts of sediment runoff from the catchment on marine habitat dynamics. Previous studies linking land and the ocean have used simple distance-based relationships between sediment load and marine habitat metrics, such as ‘relative condition’ of coral reefs [e.g., 17, 31]. Advancing this approach, Tulloch et al. [19] used a sediment plume model that accounted for depth, bathymetry, currents, and particle size of modelled sediment runoff [28] to quantify reduction in relative coral condition due to sediment. Here, we apply a model that uses spatial empirical time series data of water clarity to estimate habitat suitability for light-dependent species; it would be interesting to compare how results vary based on the different approaches. Our approach is developed for seagrass but is applicable to other benthic marine habitats that are influenced by light availability, such as algae and coral reefs, although the link between sediment loads and the marine ecosystem would need to be modified to represent the dynamics of other ecosystems. We also tested how sensitive our model was to the functional form of the relationship between sediment loads and suitable marine habitat area by running the model with a separate linear relationship between sediment loads and suitable marine habitat area (S1 Text). Surprisingly, while the amount of seagrass habitat that can be achieved by each conservation action varies depending on which relationship is used, the optimal management action does not (S4 and S6 Figs). This is a relatively well-known finding in environmental decision theory, where uncertainties in the input parameters alter predictions but do not change the relative priority of management options [68, 69]. Finally, reductions in sediment supplies, such as those resulting from the construction of dams, negatively influence marine ecosystems such as mangroves [70]; discrepancies in ecological impacts of increases versus decreases in sediment supplies to coasts is a challenge for managers.

Despite structural and parametric uncertainty in the model, a quantitative optimisation framework that explicitly links conservation across the land–ocean interface provides a major conceptual advance. Specifically, it provides a quantifiable and repeatable structure for understanding the costs and benefits of taking different conservation actions and a transparent justification for acting either on the land or in the ocean. Thus, not only are we able to argue that marine conservation actions deliver the best outcomes for marine ecosystems, but this framework also offers a mechanistic explanation for why land-based management may be inferior: Multiple time lags separate terrestrial restoration projects from marine conservation outcomes. Although there are documented instances of land-based actions delivering measurable improvements in coastal water clarity and marine habitat extent [53], these have only materialised following delays in the effect of land-based management on runoff, further delays in the improvement of coastal water clarity, and final delays in the expansion of those marine habitats. Following decades or centuries of land- and ocean-based impacts on marine ecosystems globally [1], the challenge now is to reverse the resultant declines. Using transparent, transferable, and cost-effective approaches is critical to this process.

## Materials and methods

### Study system

The study was parameterised for seagrass meadows in Moreton Bay, Queensland, Australia, and adjacent riparian areas below dams, which are considered the primary sources of sediments to the ocean in the region [42] (Lat: -27.0–28.3; Lon: 151.9–153.4) (Fig 2). Moreton Bay is a shallow coastal embayment adjacent to Brisbane, the capital city of Queensland. It is home to 18,000 ha of seagrass comprised of 7 species, which provide grazing areas for iconic, vulnerable, and threatened species such as green sea turtles, dugongs, and migratory shorebirds. Riparian areas in the catchment have been heavily cleared since European colonisation in the mid-1800s, mainly for agriculture and urbanisation, causing ongoing increased sedimentation in riverways and marine environments [41–42, 45]. Local direct threats to seagrass are mainly from physical damage from anchoring and mooring [71].

### Model—Basic framework

We extended the dynamic landscape modelling methodology of [24] to apply to both a seascape and adjacent landscape, which are connected together by sediment runoff from degraded landscapes into the ocean (Fig 1). Cleared terrestrial habitat increases sediment loads, which reduces water clarity in the adjacent ocean. The resulting decrease in light reaching the seafloor reduces the area suitable for light-dependent species. Model parameters were obtained from a variety of sources, including raster or shapefile spatial datasets (see below and S1 Table), but the dynamic landscape model is not spatially explicit. See below for additional details.

### Initial conditions

Each area of land at time *t* is classified as being in 1 of 4 states: intact and unprotected, *A(t)*; intact and protected, *P(t)*; degraded or cleared, *C(t)*; or undergoing restoration, *R(t)*, with the total amount in each state described as a proportion of the landscape, and with *A(t)* + *P(t)* + *C(t)* + *R(t)* = 1 at all times (Fig 1). The landscape area is constrained to riparian habitats, because those are the major determinant of sediment input to the ocean in Queensland and the primary target of current restoration projects [42, 72, 73]. The seascape is split into suitable habitat (sufficient light, soft sediments, and suitable wave energy, based on [52]) and unsuitable (*U*_*S*_*)* habitat. While the total area is constant, the amount of each habitat changes in each time step based on sediment loads. The area that is suitable for seagrass is divided into the same categories as on land, but denoted by the subscript *S*, with *A*_*S*_*(t)* + *P*_*S*_*(t)* + *C*_*S*_*(t)* + *R*_*S*_*(t)* = 1 at all times. These categories can be considered available and suitable, protected and suitable, etc. When an increase in sediment causes a decrease in suitable habitat, that decrease is taken in the appropriate proportions from each category of suitable habitat, which then becomes unsuitable. When sediment decreases allow for an increase in suitable habitat, this newly suitable habitat is added to the cleared and suitable state (*C*_*S*_*(t)*). Transitions between habitat categories are determined by 4 rates (degradation and revegetation on land and in the ocean) and 6 processes (restoration and protection on land and in the ocean, expansion in the ocean, and change in suitable habitat area in the ocean), which are described below.

### Relationship between terrestrial and marine habitat state

A major challenge to integrated land–sea planning is quantifying the relationship between actions undertaken on land and their effects on the marine environment. For seagrass meadows in Moreton Bay, this relationship is primarily defined by the effects of terrestrial sediment runoff on the amount of illuminated seafloor available in the ocean. We used an ‘action–response’ curve [sensu 51] describing the relationship between sediment loads and seagrass-suitable habitat area calculated in (S1 Text), which in turn uses the habitat distribution model published in [52], monthly water quality data, and monthly sediment load data (S1 Text, S10 Data). This relationship predicts the area of habitat that is suitable for seagrass in each year based on the sediment loads delivered from the catchment in the previous year. This approach provides a simplification of a complex system, whereby sediment distribution and resuspension are affected by sediment composition, rainfall, and oceanographic processes, among other factors. Factoring in spatially explicit hydrodynamic modelling of sediment distribution would be an important next step to this research.

### Changes in area of seagrass suitable habitat

Transitions in the area of suitable and unsuitable marine habitat are determined by the amount of intact land (*A* + *P*) in the previous time step, which affects the quantity of sediment delivered to the ocean and the area of habitat available for light-dependent marine species, like seagrass. If the area of suitable habitat is greater in *t* than in *t*−1, then the newly suitable habitat area is added to the Cleared (*C*_*s*_) fraction, since this habitat would not contain seagrass at the outset. If the area of suitable habitat decreases in *t* compared to *t*−1, then habitat is removed proportionally from *A*_*S*_, *P*_*S*_, *C*_*S*_, and *R*_*S*_.

### Conservation actions

Information on the definitions of the 4 conservation actions (marine restoration, marine protection, land restoration, and land protection), as well as on their costs and probabilities of success, are given in the Results, S1 Text and S1 Table.

### Rates, processes, and initial conditions

Here, we provide a summary of the model parameters describing the rates, processes, and initial conditions. Further information is given in S1 Text and S1 Table.

#### Sediment loads

At present, an average of 280,000 tonnes of sediment are delivered to Moreton Bay each year [74] from a catchment containing 20% intact riparian areas [42]. Pre-European colonisation, the annual sediment load was approximately 100,000 tonnes [75]. Based on these values and a linear relationship between the percent of intact riparian areas and sediment load, we estimated that the average yearly sediment load would be 350,000 tonnes if the catchment were completely cleared. This does not account for temporal variation in sediment delivery driven by climate change and environmental stochasticity, which is an important area of future research.

#### Habitat area in each state

Initial conditions for the area of cleared and available riparian and seagrass habitats and the area of marine habitat unsuitable for seagrass were derived from [42, 52, 76, 77]. The area of protected riparian habitats was derived from [76]. There are currently over 200 environmentally friendly moorings in Moreton Bay protecting an estimate 20 ha of seagrass. We assumed that none of the seagrass or riparian habitats were in restoring condition in the initial timestep. The total area of seagrass which could be in restoring condition at any time was limited to 0.1% of the existing meadow to reflect logistical limitations on our ability to restore seagrass. The upper limit on the area of protected seagrass habitat was 8.8%, because of the 33,520 ha suitable for seagrass in Moreton Bay, 2,966 ha are appropriate for moorings (based on results in [52]).

#### Transition rates

The current rates of decline for riparian and seagrass habitats were 0.75% per year (derived from [76, 78, 81] using GIS analysis) and 0.5% per year [62], respectively. The rates of revegetation (the time lags before newly restored habitat become intact habitat) were 3 and 10 years for seagrass and riparian habitats, respectively [47, 82]. In reality, sediment loads take variable lengths of time to decline and, in some instances, increase following restoration [66, 83]. The rate of expansion of seagrass habitats was 1.13% per year, based on the observed rate of expansion of seagrass in Tampa Bay, Florida, over 22 years, achieved by reductions in nitrogen from land [53]. Tampa Bay has a similar climate and size to Moreton Bay, and we therefore expect that seagrass in Moreton Bay could respond similarly to a reduction in land-based impact. However, there is considerable uncertainty in this parameter (S2 Table), and this estimate is conservative. Accordingly, we examine how our decision-making varies under various estimates for rate of seagrass expansion (See Fig 4).

### Scenarios

We run the model for 2 scenarios. For both scenarios, we run 4 allocation simulations, where the budget is allocated to each of the actions in isolation. Each simulation expends a budget of $50 million per year over 30 years in 0.1-year time intervals, for a total of $1.5 billion (not accounting for inflation and discount rates). By comparison, it will cost $5–$10 billion over 10 years to mitigate sedimentation issues in the Great Barrier Reef catchments [84]. The time horizon of 30 years aligns with other management policies aimed at mitigating sediment issues, e.g., the ‘Reef 2050 Long-term Sustainability Plan’ for the Great Barrier Reef [34]. All results are standardised to the outcomes achieved with no investment. For the first scenario, we invest the budget according to the parameters that we believe best describe the system. For the second scenario, we run a sensitivity analysis to examine uncertainty or variability in some of the key parameters identified in the model development process. Specifically, the model is run for rates of marine ecosystem decline and expansion varying by 0%–7% and 0%–10% per year, respectively. This approach is replicated for 4 different ecological and resource use contexts, encompassing historic land clearing extent = 20 or 80% and rate of land clearing = 0.75 or 7% per year. Further sensitivity analyses on the effects of time lags in restoration success, costs of actions, and the maximum area suitable for marine restoration or protection are provided in the Supporting Information and are outlined in S3 Table.

### Model equations

At the beginning of each time step, the area of suitable marine habitat is calculated according to the area of intact (*A* + *P*) habitat on land. Next, changes in land and ocean habitat in each time step *Δt* are modelled as described by the following equations, where *B* is the annual budget, *S* is the area of marine habitat, and *L* is the area of land habitat. Subscripts to *S* and *L* describe the proportional areas of different habitat categories transitioning between fractions. A representative model output describing the land–sea dynamics through time is provided in S7 Fig, and additional detail is given in S1 Text.

#### Marine restoration

The area of marine habitat transitioning from the cleared (*C*_*S*_*(t)*) to the restoring fraction (*R*_*S*_*(t)*) at time *t* as a result of restoration (*S*_*R*_*(t)*) is:

$$S_{R}=\Delta t.F_{SR}.B/C_{SR}$$

where *F*_*SR*_ is the feasibility (percentage) of marine restoration, and *C*_*SR*_ is the per unit cost of marine restoration.

#### Marine revegetation

The area of marine habitat transitioning from the restoring (*R*_*S*_*(t)*) to the protected fraction (*P*_*S*_*(t)*) due to revegetation (*S*_*V*_) is:

$$S_{V}=\Delta t.g_{S}.R_{S}\left(t\right)$$

where *g*_*s*_ is the proportional marine revegetation rate in percent per year and *R*_*S(t)*_ is the proportional area of marine habitat that has undergone restoration actions.

#### Marine protection

The area of marine habitat transitioning from the available *A*_*S(t)*_ to the protected (*P*_*S*_*(t)*) fraction (*S*_*P*_) is:

$$S_{P}=\Delta t.B/C_{SP}$$

where *C*_*SP*_ is the per unit cost of marine protection.

#### Marine degradation

The area of marine habitat transitioning from the available *A*_*S*_*(t)* to the cleared *C*_*S*_*(t)* fraction (*S*_*D*_) is:

$$S_{D}=\Delta t.d_{s}.A_{S}\left(t\right)$$

where *d*_*s*_ is the proportional rate of marine habitat degradation in percent per year and *A*_*S*_*(t)* is the proportional area of intact unprotected marine habitat at time *t*.

#### Marine expansion

The area of marine habitat transitioning from the cleared (*C*_*s*_*(t)*) to the available (*A*_*s*_*(t)*) fraction at time *t* due to expansion into suitable habitat (natural recovery, *S*_*E*_) is:

$$S_{E}=dt.g_{s}.\left(A_{s}\left(t\right)+P_{s}\left(t\right)\right)$$

where *g*_*s*_ is the proportional expansion rate of seagrass in percent per year, *A*_*S*_*(t)* is the proportional area of intact unprotected marine habitat at time *t*, and *P*_*S*_*(t)* is the proportional area of protected seagrass at time *t*. Note that *S*_*E*_ is limited by the amount of cleared marine habitat.

#### Land restoration

The area of land changing from the cleared (*C(t)*) to the restoring (*R(t)*) fraction at time *t* as a result of restoration actions (*L*_*R*_) is:

$$L_{R}=\Delta t.F_{LR}.B/C_{LR}$$

where *F*_*LR*_ is the feasibility (percentage) of land restoration and *C*_*LR*_ is the cost of land restoration.

#### Land revegetation

The area of land transitioning from the restoring (*R(t)*) to the protected (*P(t)*) fraction due to revegetation (*L*_*V*_) is:

$$L_{V}=\Delta t.g_{L}.R\left(t\right)$$

where *g*_*L*_ is the proportional revegetation rate of land in percent per year and *R(t)* is the proportional area of restoring habitat on land at time *t*.

#### Land protection

The area of riparian habitat transitioning from the available *A(t)* to protected *P*_*S*_*(t)* fraction (*L*_*P*_) is:

$$L_{P}=\Delta t.B/C_{P}$$

where *C*_*P*_ is the cost of land protection.

#### Land degradation

The area of land transitioning from the available *A(t)* to the cleared *C(t)* fraction due to degradation on land (*L*_*D*_) is:

$$L_{D}=\Delta t.d_{l}.A\left(t\right)$$

where *d*_*l*_ is the proportional rate of land degradation in percent per year and *A(t)* is the proportional area of intact land at time *t*.

In each time step, after accounting for changes in marine habitat areas caused by sedimentation from the previous time step, the habitat areas described above were added or subtracted from the fractions within the land- and sea-scape to achieve the new land- and sea-scape for that time step according to the following equations:

Transitions in the sea:

$$R_{S\left(t\right)}=R_{S\left(t-1\right)}+S_{R}-S_{V}$$

$$C_{S\left(t\right)}=C_{S\left(t-1\right)}-S_{R}+S_{D}-S_{E}$$

$$P_{S\left(t\right)}=P_{S\left(t-1\right)}+S_{V}+S_{P}$$

$$A_{S\left(t\right)}=S_{S\left(t-1\right)}-S_{P}-S_{D}+S_{E}$$

Transitions on land:

$$R_{\left(t\right)}=R_{\left(t-1\right)}+L_{R}-L_{V}$$

$$C_{\left(t\right)}=C_{\left(t-1\right)}-L_{R}+L_{D}$$

$$P_{\left(t\right)}=P_{\left(t-1\right)}+L_{V}+L_{P}$$

$$A_{\left(t\right)}=S_{\left(t-1\right)}-L_{P}-L_{D}$$

## Supporting information

S1 Fig Sensitivity analysis. Intact seagrass area obtained using a cap of (A) 0.1% and (B) 1% of the existing seagrass meadows which may be in “restoring” condition in a given year.
(TIF)

S2 Fig Sensitivity analysis. Effect of the rate of revegetation of seagrass and riparian habitats following restoration actions on the optimal conservation decision after 30 years. Results are reported for two rates of seagrass expansion: A) 1% yr^-1^; and B) 5% yr^-1^.
(TIF)

S3 Fig Sensitivity analysis. Effect of the costs of seagrass and riparian restoration on the optimal conservation decision after 30 years. Results are reported for two rates of seagrass expansion: A) 1.13% yr^-1^; and B) 5% yr^-1^.
(TIF)

S4 Fig Sensitivity analysis. Effect of seagrass expansion and decline rates on the (A,B) optimal action, and (C,D) relative area of seagrass habitat, compared to a no investment strategy, after 30 years. Two functional relationships between sediment load and habitat area were used: (A,C) linear relationship; and (B,D) convex relationship. The convex relationship was generated using a habitat distribution model and time varying sediment loads (1). The linear relationship was derived using a linear approximation instead of a polynomial fit.
(TIF)

S5 Fig Sensitivity analysis. Effect of seagrass decline and expansion rates on the optimal conservation strategy if the objective is to maximise the value of ecosystem services returned by both seagrass and riparian habitats over a 30 year investment period.
(TIF)

S6 Fig Sensitivity analysis. Results obtained using a linear relationship between sediment load and seagrass area, compared to a convex relationship used in Fig 3. Areas of A) habitat suitable for seagrass; B) protected seagrass; C) intact seagrass; and D) tons per year of sediment run-off. Values are standardised to the values achieved with no investment.
(TIF)

S7 Fig Model dynamics. Dynamics of the land- and sea-scape model of seagrass meadows (ocean) and riparian habitats (land) over 30 years based on the actions of restoration or protection in both systems.
(TIF)

S1 Table Model parameters. Model parameters used to assess the cost effectiveness of conservation actions (restoration or protection) taken on land or sea to maximize extent of marine habitat (seagrass) in Moreton Bay, Southeast Queensland, Australia. Justification for the parameterisation is provided in the supplemental methods.
(XLSX)

S2 Table Seagrass expansion rates. Studies from which estimates of areal expansion of seagrass beds were obtained. *: study measured gap size.
(XLSX)

S3 Table Summary of sensitivity analyses. Table outlining sensitivity analyses.
(XLSX)

S1 Text Supplementary methods. (DOCX)

S1 Data Data used to generate Fig 3. (XLS)

S2 Data Data used to generate Fig 4. (XLS)

S3 Data Data used to generate S1 Fig. Data used to generate S1 Fig panel B. Data for S1 Fig panel A are found in in S1 Data.
(XLS)

S4 Data Data used to generate S2 Fig. (XLS)

S5 Data Data used to generate S3 Fig. (XLS)

S6 Data Data used to generate S4 Fig. (XLS)

S7 Data Data used to generate S5 Fig. (XLS)

S8 Data Data used to generate S6 Fig. (XLS)

S9 Data Data used to generate S7 Fig. (XLS)

S10 Data Total suspended sediment (TSS) data output from the Source model of Southeast Queensland, Australia. (XLSX)

S1 Code Matlab code. (DOCX)

S2 Code Input parameters for Matlab code. The supplemental Matlab code calls the parameter values entered in this spreadsheet.
(XLSX)

## Abbreviations

MPA: Marine Protected Area
