# Supplementary figures and images for: Simple rules can guide whether land- or ocean-based conservation will best benefit marine ecosystems
Source: PLoS Biol. 2017 Sep 6;15(9):e2001886. doi: 10.1371/journal.pbio.2001886 (PMC5587113; doi:10.1371/journal.pbio.2001886)

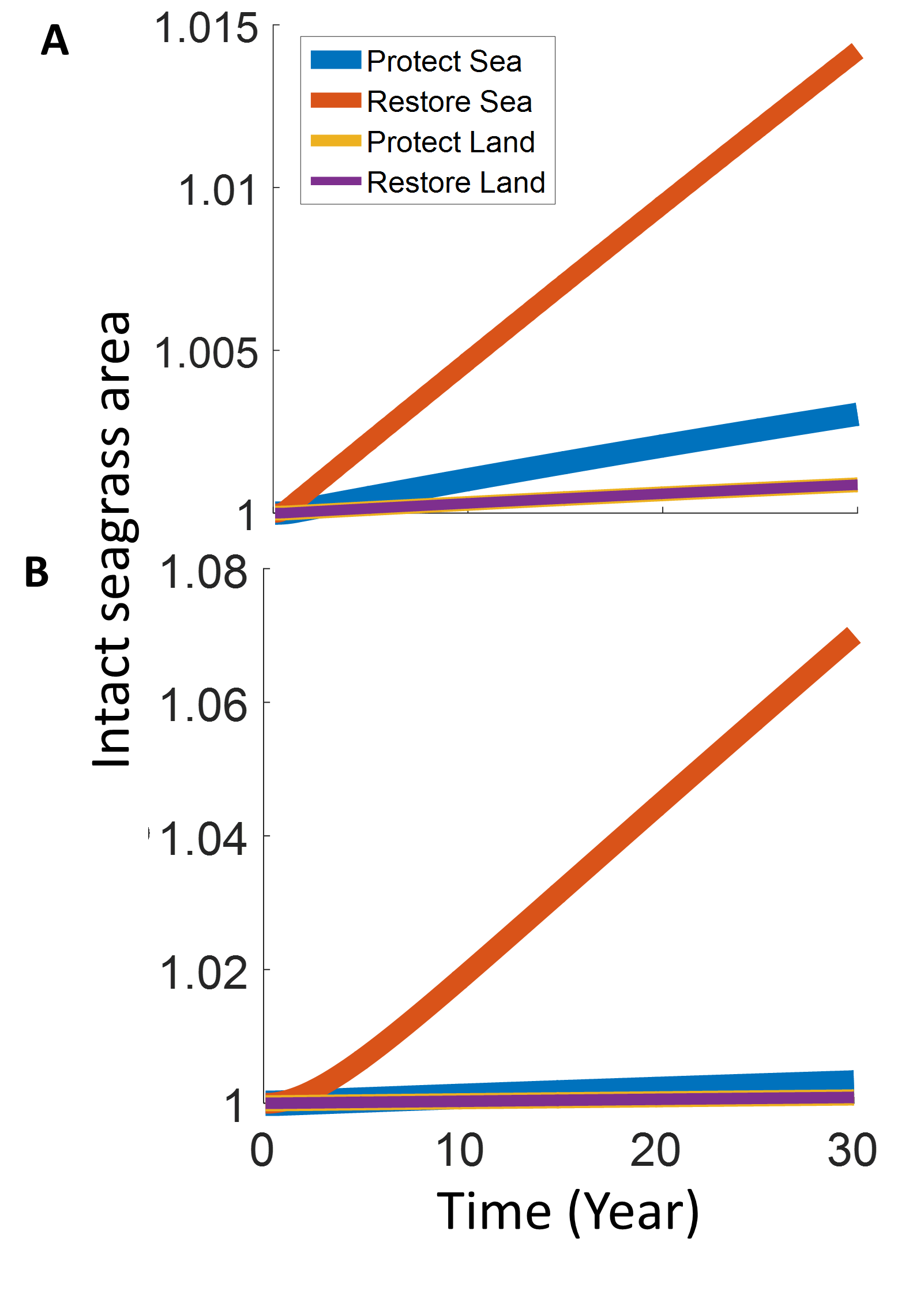

Supplement: S1 Fig — Intact seagrass area obtained using a cap of (A) 0.1% and (B) 1% of the existing seagrass meadows which may be in “restoring” condition in a given year. (TIF) [file pbio.2001886.s001.TIF]

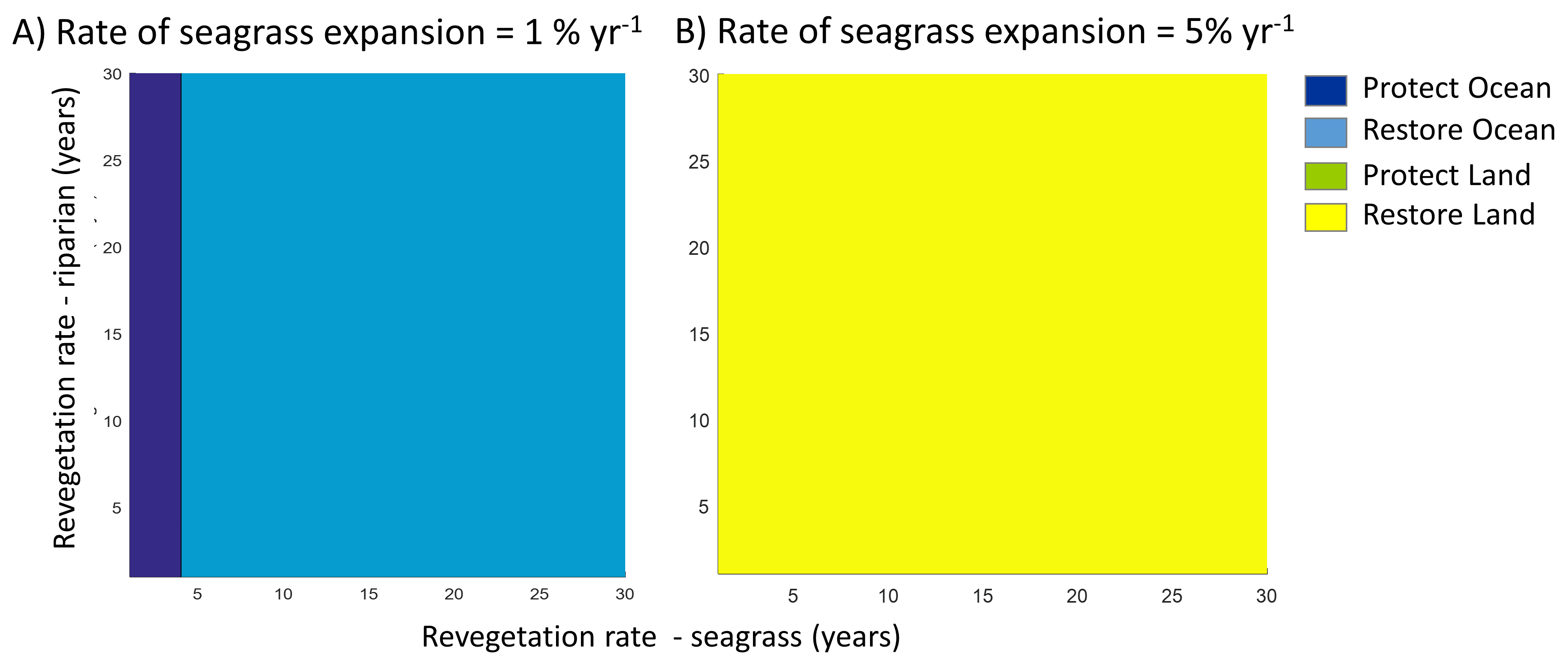

Supplement: S2 Fig — Effect of the rate of revegetation of seagrass and riparian habitats following restoration actions on the optimal conservation decision after 30 years. Results are reported for two rates of seagrass expansion: A) 1% yr-1; and B) 5% yr-1. (TIF) [file pbio.2001886.s002.TIF]

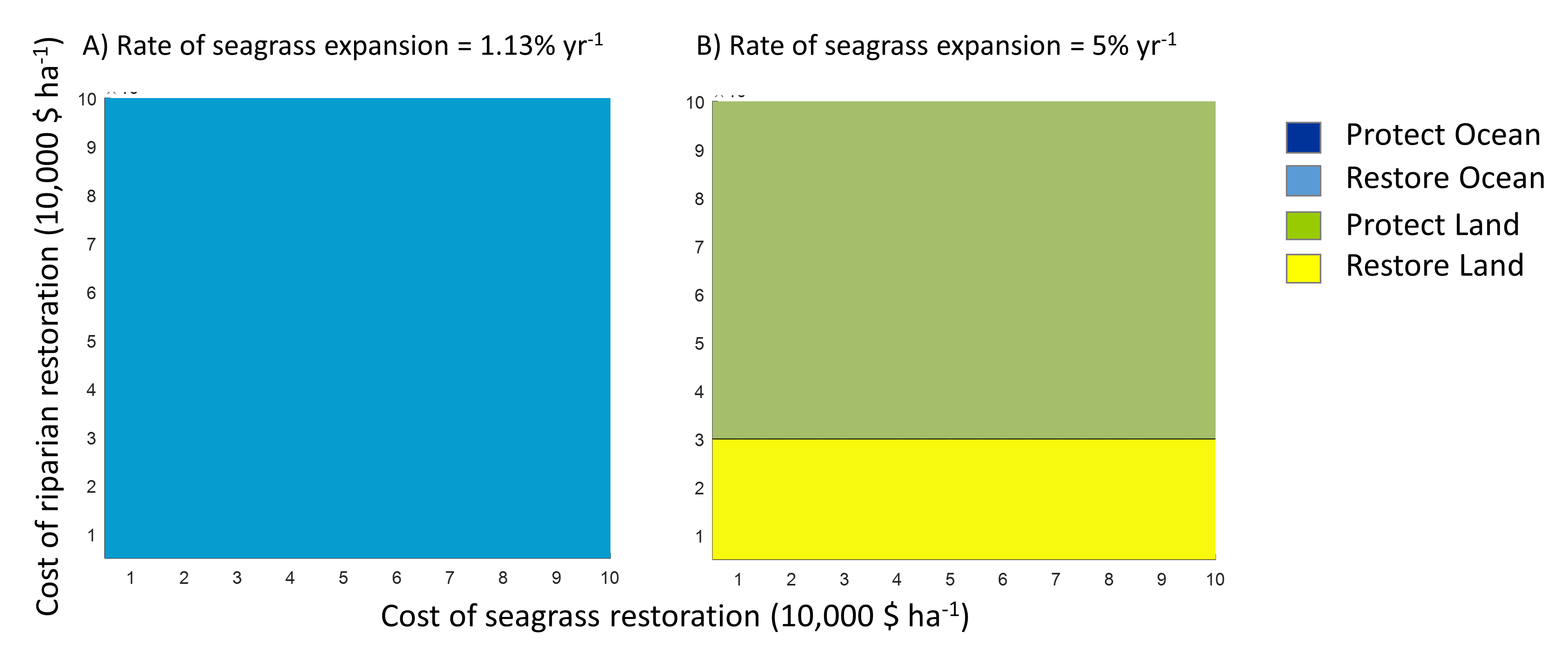

Supplement: S3 Fig — Effect of the costs of seagrass and riparian restoration on the optimal conservation decision after 30 years. Results are reported for two rates of seagrass expansion: A) 1.13% yr-1; and B) 5% yr-1. (TIF) [file pbio.2001886.s003.TIF]

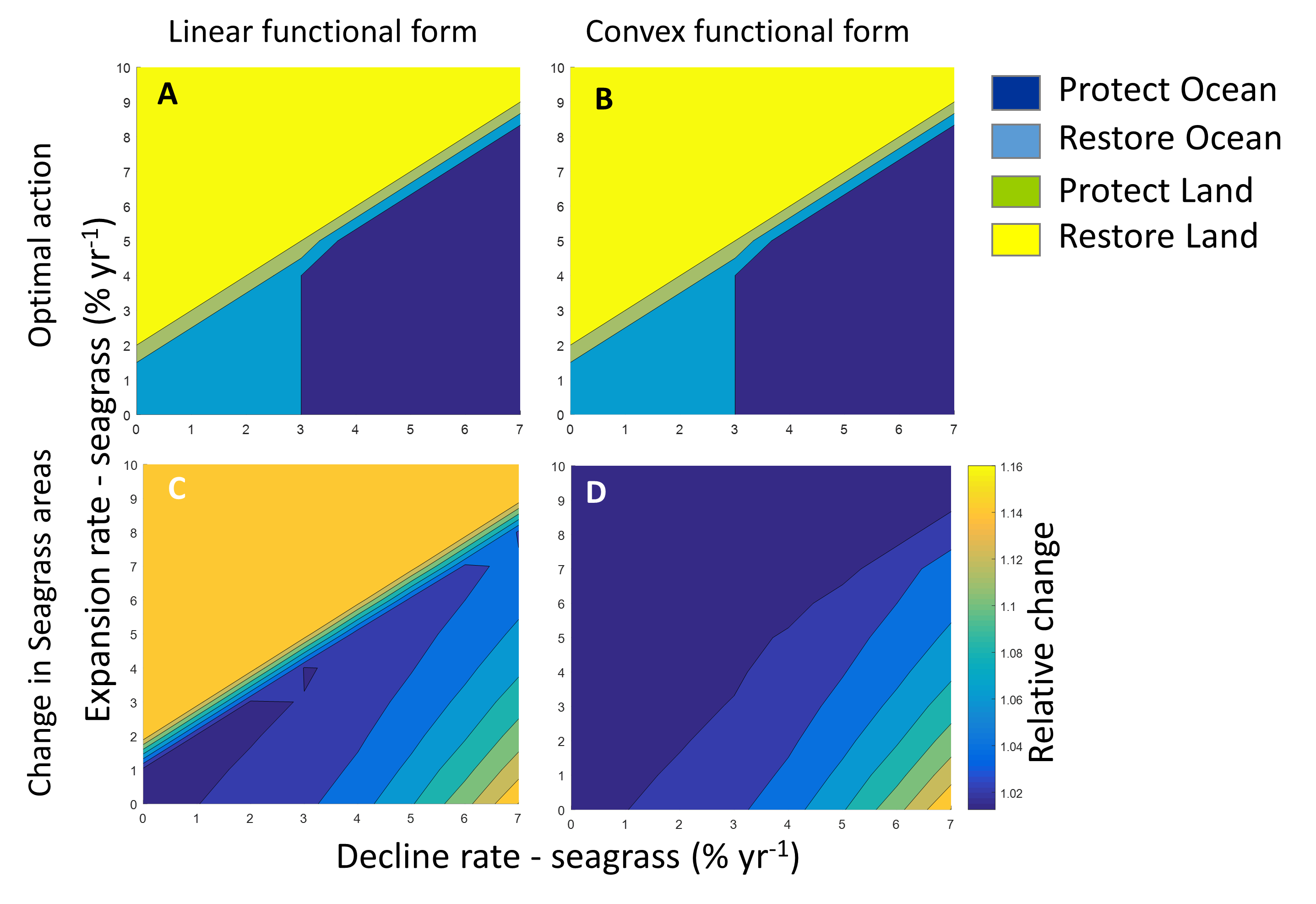

Supplement: S4 Fig — Effect of seagrass expansion and decline rates on the (A,B) optimal action, and (C,D) relative area of seagrass habitat, compared to a no investment strategy, after 30 years. Two functional relationships between sediment load and habitat area were used: (A,C) linear relationship; and (B,D) convex relationship. The convex relationship was generated using a habitat distribution model and time varying sediment loads (1). The linear relationship was derived using a linear approximation instead of a polynomial fit. (TIF) [file pbio.2001886.s004.TIF]

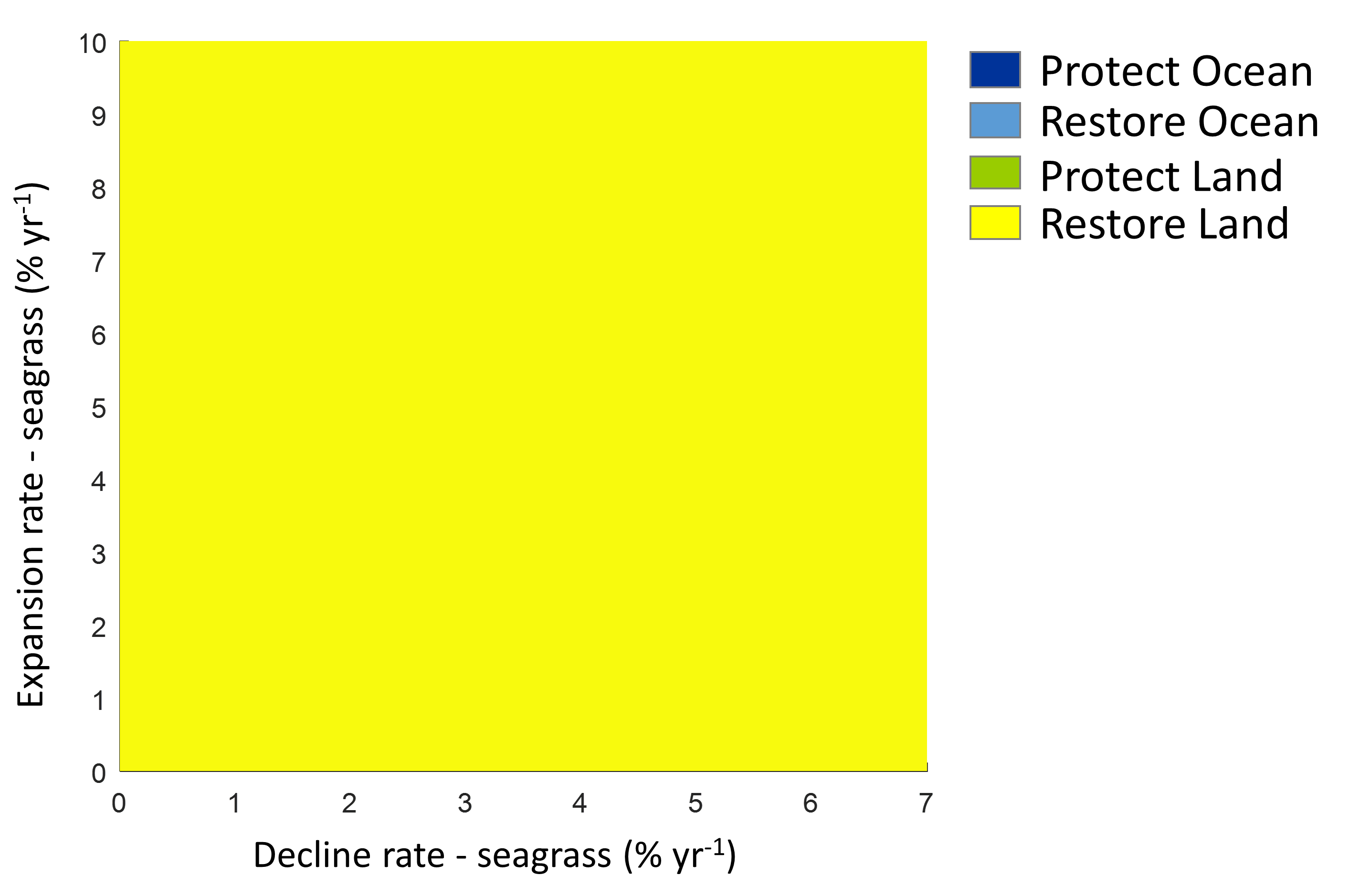

Supplement: S5 Fig — Effect of seagrass decline and expansion rates on the optimal conservation strategy if the objective is to maximise the value of ecosystem services returned by both seagrass and riparian habitats over a 30 year investment period. (TIF) [file pbio.2001886.s005.TIF]

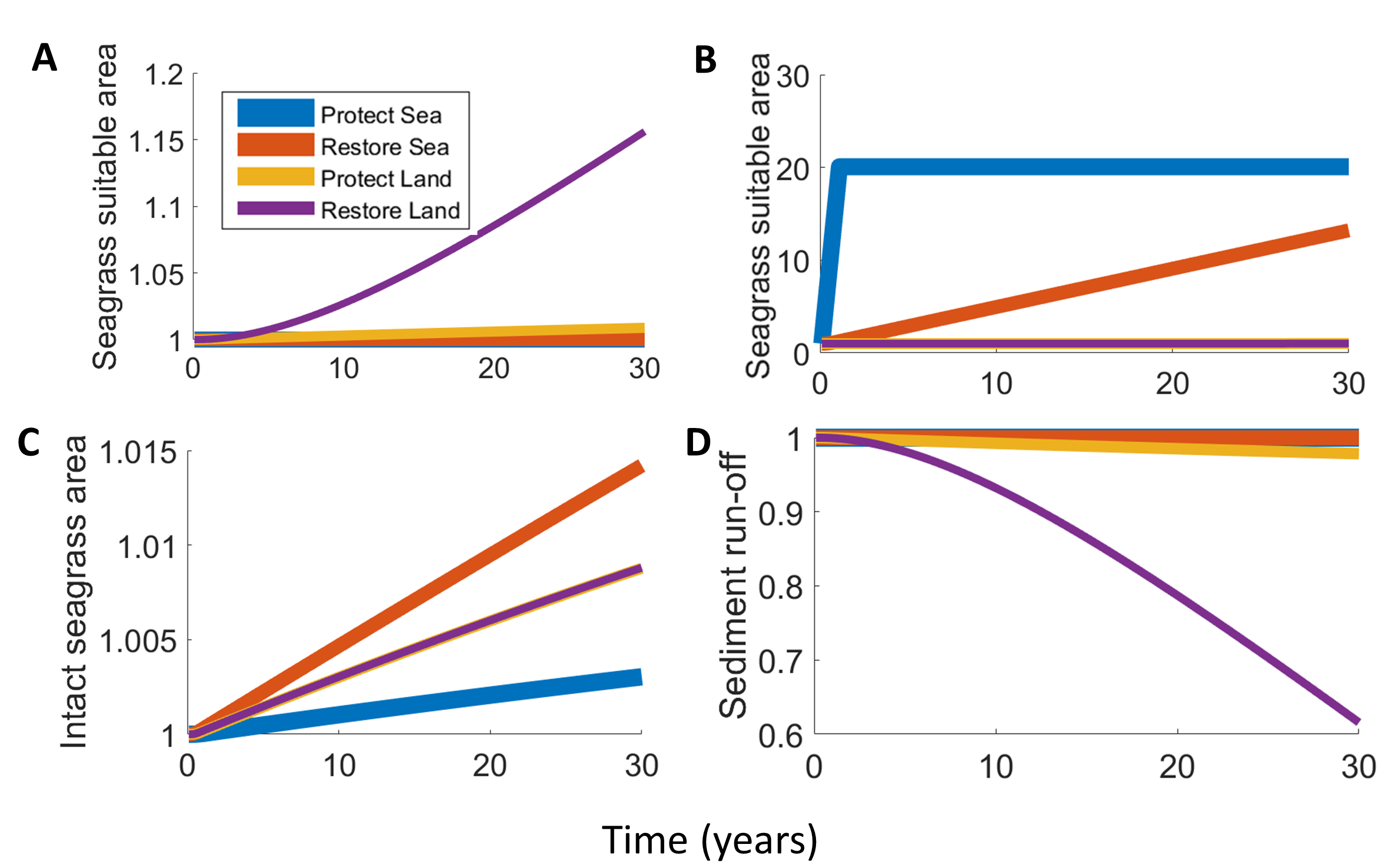

Supplement: S6 Fig — Results obtained using a linear relationship between sediment load and seagrass area, compared to a convex relationship used in Fig 3. Areas of A) habitat suitable for seagrass; B) protected seagrass; C) intact seagrass; and D) tons per year of sediment run-off. Values are standardised to the values achieved with no investment. (TIF) [file pbio.2001886.s006.TIF]

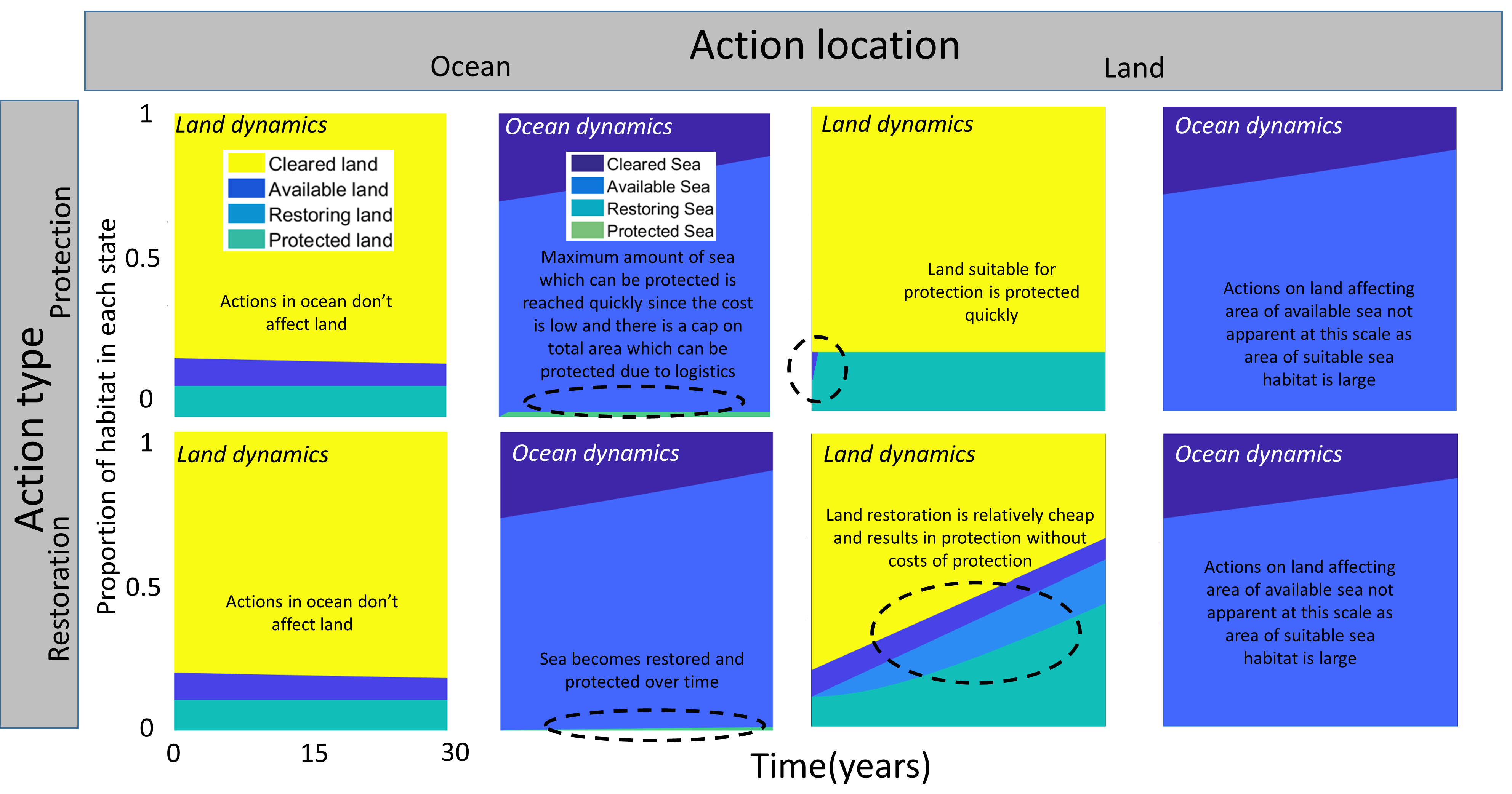

Supplement: S7 Fig — Dynamics of the land- and sea-scape model of seagrass meadows (ocean) and riparian habitats (land) over 30 years based on the actions of restoration or protection in both systems. (TIF) [file pbio.2001886.s007.TIF]
