# Supplementary material for: Simple rules can guide whether land- or ocean-based conservation will best benefit marine ecosystems
Source: PLoS Biol. 2017 Sep 6;15(9):e2001886. doi: 10.1371/journal.pbio.2001886 (PMC5587113; doi:10.1371/journal.pbio.2001886)
Supplement: S1 Text — (DOCX) [file pbio.2001886.s011.docx]

**Supplemental methods to:** Simple rules can guide whether land or ocean based conservation will best benefit marine ecosystems

Megan I. Saunders^1,2,3,4,5*^, Michael Bode^2,6^, Scott Atkinson^1,2^, Carissa J. Klein^1,2,3^, Anna Metaxas^7^, Jutta Beher^1,2^, Maria Beger^1,2,8^, Morena Mills^1,2,9^, Sylvaine Giakoumi^1,2,10^, Vivitskaia Tulloch^1,2^, Hugh P. Possingham^1,2,11^

**Affiliations:**

1. Centre for Biodiversity and Conservation Science, The University of Queensland, St. Lucia, Australia 4072
2. Australian Research Council (ARC) Centre of Excellence in Environmental Decisions, University of Queensland, St Lucia, 4072 Australia
3. School of Earth and Environmental Sciences, The University of Queensland, St. Lucia, Australia, 4072
4. The Global Change Institute, The University of Queensland, St Lucia, Australia, 4072
5. School of Chemical Engineering, The University of Queensland, St. Lucia, Australia 4072
6. Australian Research Council Centre of Excellence for Coral Reef Studies, James Cook University, Townsville, Australia, 4811.
7. Department of Oceanography, Dalhousie University, Halifax, Nova Scotia, Canada, B3H 4R2
8. School of Biology, University of Leeds, Leeds, LS2 9JT, UK
9. Department of Life Sciences, Imperial College London, Silwood Park Campus, Buckhurst Road, Ascot, Berkshire SL5 7PY, UK
10. Université Côte d’Azur, CNRS, FRE 3729 ECOMERS, Parc Valrose, 28 Avenue Valrose, 06108 Nice, France
11. The Nature Conservancy, Arlington VA, USA, 22203

* m.saunders1@uq.edu.au

**Relationship between sediment load and seagrass suitable habitat**

**Sediment loads.** Terrestrial sediment input to the coast was estimated using modelled daily sediment discharge data from the Source modelling suite [1] using the SIMHYD rainfall runoff model [2] for the catchments of Southeast Queensland. Input data included gridded daily rainfall and potential evapotranspiration at 5 x 5 km resolution [3] and land uses (1:50,000) [4]. Data exported from the model included total suspended solids (TSS, in Tonnes, hereafter ‘sediment’) outputs for rivers draining into Moreton Bay for Jan 1980 to June 2014. Sediment data were summed across the rivers and aggregated monthly. Sediment data were produced by T. Weber, Alluvium Consulting, for Healthy Land and Water.

**Impact of sediment load on benthic light availability.** Water clarity was assumed to be influenced by the distance to open ocean, distance to river mouths, and water depth. River mouths were located using [5], and the location of open ocean was defined by the 30m depth contour [6]. Secchi depth data sampled at 51-91 sites in 2000-2013 were obtained from Healthy Waterways ([http://www.ehmp.org](http://www.ehmp.org/)). Secchi depth (*Z_SD_*) was modelled by fitting a Linear Model to Secchi depth, with sediment (*S*), depth (*z*), distance to rivers (*D_R_*), and distance to open ocean (*D_O_*) as predictor variables based on [7]. Model coefficients were used to predict *Z_SD_* at all other locations for each month from 2000 to 2013 using the data layers for Secchi depth and water depth. Irradiance *I* (W m^2^ s^-1^) at depth *z* of a given location depends upon water clarity and depth (m) [8]:

$I\left( Z \right)=I_{0}e^{-k_{d}Z}$ (1)

where *I_0_* is surface irradiance (W m^2^ s^-1^) and *K_d_* is the diffuse attenuation coefficient (m^-1^). Percent light available at water depth *z* was estimated by *I(z)*/*I_0_*. *K_d_* is a function of Secchi depth (*Z_SD_*) and the constant *λ* (estimated to be 1.7 [9]):

$K_{d}=\frac{\lambda}{Z_{SD}}$ (2)

**Habitat distribution model.** The presence or absence of seagrass (from [10]) was predicted by fitting a Generalised Linear Model (GLM) assuming a binomial distribution to the input data, with the logarithm of percent surface irradiance (mean from 6 months prior) (‘light’), significant wave height (‘waves’) [11, 12], and the interaction between light and waves, as predictor variables (based on [7]). A threshold cut-off value was calculated to be 0.22 by maximising kappa. The habitat distribution model was used to create maps of probability of seagrass presence from Feb 2000 to Mar 2013. In each grid cell the probability of seagrass occurring (*p_i_*) was determined by [13]:

$p_{i}=\frac{e^{g\left( x_{1} \right)}}{1+e^{g\left( x_{i} \right)}}$ (3)

where *g(x_i_)* is the linear predictor fitted by the logistic regression. Coefficients of the model were estimated using seagrass and sediment data from 2004, and the fitted model was used to predict probability of seagrass presence in other locations and time periods.

**Relationship between sediment loads and seagrass suitable area.** The area of seagrass suitable habitat in each month was calculated by summing the number of 1 ha cells where seagrass was predicted to occur for each month and then averaging values over the wet season of each year (Nov to March) as a conservative estimate of the habitat suitability over the year from July to June. The effect of sediment load on seagrass suitable habitat area on annual time scales was estimated using a linear model, with habitat area as the response variable and annual sediment^2^ as the predictor variable(s), selected by minimizing BIC.

**Relationship between sediment load and area of seagrass suitable habitat.** We found that the functional relationship between sediment supply to the ocean (*S_t_* ) and hectares of seagrass suitable habitat (*H_t_*) at time *t* takes the form:

$$H_{t}={-5.66}^{-9}\times S_{t}^{2}+23600$$

To test the implications of this functional form we also use a linear relationship based around similar values in some of the sensitivity analyses.

$$H_{t}=-0.0267\times S_{t}+26667$$

**Justification of model parameters**

**Sediment loads.** At present 280,000 tonnes of sediment are delivered on average to Moreton Bay each year [14] from a catchment containing 20% intact riparian areas [15]. Pre-European colonisation, the annual sediment load was approximately 100,000 tonnes [16]. Based on these values and a linear relationship between the percent of intact riparian areas and sediment load, the average yearly sediment load if the catchment were completely cleared would 350,000 tonnes. This does not account for climatic variation in sediment delivery, which is an important area of future research. For example, in 2011 heavy sustained rainfall in SE Qld resulted in floods delivering 1,000,000 tonnes of sediment to Moreton Bay, with far reaching impacts on the marine environment [17].

**Conservation Actions – Definition, Cost, Probability of success, and Limits**

Land protection was defined as purchasing intact habitat on land and zoning it as a nature reserve, at a cost of $3530 ha^-1^. The sale price of land is based on a statistical model developed by Martin Taylor at World Wildlife Fund [18], which takes into account unimproved land value and market value. The minimum and maximum values were $135 ha^-1^ and $21,042 ha^-1^, respectively. The values used were specific to the Lockyer Valley, where 70% of the sediment that is deposited in Moreton Bay is eroded [3]. Mean predicted sale price for all "natural or relatively natural land" which is not already in a national park was calculated according to the Queensland Land Use Shapefile of Lockyer from 2012 [8]. This includes land that is grazed but is "relatively natural", and may therefore may be a large underestimation of costs, as some restoration actions would be required to increase the quality of land to national park status. We assume that the area required from the land parcels which border the river can be purchased and that these areas do not contain buildings.

Land restoration was defined as the process of stabilizing banks and planting trees in cleared areas, at a cost of $17,310 ha^-1^ and an estimated probability of success of 50% (based on data in Fig 1 and Table 5 in [19]; J. Omara, SEQ Catchments, pers. Comm.). The probability of success estimate for riparian restoration was estimated for riparian restoration projects in Southeast Queensland and is broadly in line with estimates of marine restoration success of 38-65% [20]. After restoration it was assumed the land would be protected from further degradation. This could occur, for example, through schemes to help landowners improve farming practices, whereby they do not give up ownership of the land, but agree to not degrade it in future.

Marine protection was defined as the implementation of physical structures to prevent damage to seagrass plants caused by anchoring, at a cost of $131,000 ha^-1^ [21]. For Moreton Bay, these structures include Environmental Friendly Moorings (EVM) which protect seagrass from direct damage caused by anchors and conventional moorings. The system is comprised of a pivoting raised arm attached to a Helix screw anchor point. Each mooring costs $2500 for hardware, $300 for installation, plus $300 yr^-1^ maintenance costs over the mooring lifespan of 15 years. For a 30 year time period the mooring would need to be replaced once. Each EVM protects 0.0895 ha of seagrass. Summing the costs per location for 30 years and assuming that each mooring protects 0.0895 ha of seagrass gives a total cost for marine protection of $131,843 ha^-1^. The amount of marine habitat suitable for protection was capped at 8.8% of the suitable habitat for seagrass, because 2,966 ha in Moreton Bay are considered appropriate for moorings, which equals to 8.8% of the 33,520 ha supporting seagrass.

Marine restoration was defined as planting seagrass plugs from donor sites at a cost of $418,000 ha^-1^ and a probability of success of 38% [20]. The cost of seagrass restoration was based on the average value for seagrass restoration projects from a meta-analysis of marine restoration literature [20]. The amount of marine habitat suitable for restoration was capped at 0.1% of existing meadows each year, allowing for limitations to the success of large scale restoration, and to minimize damage to the existing meadows which would need to provide source material. This equates to 17 ha over the restoring period of 3 years, or 5.6 ha per year - well within the range of restored seagrass habitat achieved in a meta-analysis of published seagrass restoration trials [20], and without using the entire budget in each year. If a less conservative estimate of 1% for the area which can be in restoring condition is assumed, then a greater amount of seagrass can be achieved over the project duration (**S5_Fig**).

There was no cap imposed on the area of cleared land which could be restored or protected. All costs have been converted to 2015 USD by converting to USD and then adjusting to 2015 according to inflation.

**Initial conditions for areas of habitat**

The areas of habitat on land and ocean in each of the habitat categories used as the initial conditions for the model were obtained by GIS analysis in ArcGIS, through values from the literature, and from personal communications.

*Land.* There are 47,960 km of stream network in Southeast Queensland, of which 17,095 km are below the dams [15]. Assuming that riparian habitats extend 15 m on each side of the streams, giving a 30 m buffer zone, this gives a riparian habitat area of 74,631 ha below the dams. Olley et al. [15] report that 81% of the riparian vegetation below the dams has been cleared, leaving that 19% intact remnant vegetation. We conducted a GIS analysis using data from the Statewide Landcover and Trees Study [22], to determine that 53% of the vegetation in the region is protected, and 47% in unprotected. Assuming that the same proportion of protection applies to the remnant riparian protection, we calculate than 9520 ha of remnant vegetation is protected and 8442 ha unprotected. We assumed that none of the riparian vegetation was in a ‘restoring’ category (e.g. restored within the past 10 years) for the initial conditions of the model. The width of the buffer influences the results in our model – for instance, if a 10 or 100 m buffer is used then a larger or smaller proportion area of riparian habitat can be restored for a given budget, respectively. The stream length of riparian habitats below dams in southeast Queensland that would be needed to be restored to halve the sediment delivery to Moreton Bay was recently estimated at 6350 km [15].

*Sea.* The area of seagrass habitat was obtained from maps made using remote sensing published in [10] and clipped to our model domain, for a total of 16,776 ha. Of this, 20 ha are currently protected by 200 seagrass friendly moorings, giving 16,756 ha that are intact and unprotected. Data for cleared areas in the sea are more difficult to attain than for terrestrial habitats, therefore we used information from a habitat distribution model, which predicts the area of seafloor which is suitable for seagrass. Subtracting the area of potential habitat which is occupied by seagrass from the area which is suitable gives the area of cleared habitat, at 6,839 ha. As for areas on land, we assumed that none of the marine habitat was undergoing restoration at the beginning of the simulations. The area that is unsuitable for seagrass was obtained from the habitat distribution model, and estimated at 308,905 ha.

**Rates of degradation**

*Land.* The background rate of degradation on land was estimated from land-use maps to be 0.75 % yr^-1^. This was based on the mean change (in %) of woody vegetation cover in the Lockyer Creek drainage between 2008/2009 and 2011/2012 quantified using a GIS analysis of the Land Cover Change in Queensland: 2008/2009, 2009/2010, 2010/2011, 2011/2012 SLATS Reports [22-26]. The SLATS program annually maps vegetation extent for all perennial wooded vegetation using available Landsat TM/ETM+ satellite imagery. We considered areas with woody vegetation as habitat available for protection, and calculated the mean rate of change in woody vegetation cover (reported as a %) from each preceding year over the period 2008/2009 to 2010/2011.

*Ocean.* The background rate of degradation in marine ecosystems is more challenging to obtain due to limitations in mapping methodologies from remote sensing in regions with turbid water [27]. Therefore, we used a value for the rate of loss of a particular category of seagrass from a section of the study site – namely, high density seagrass from the Eastern Banks of Moreton Bay, where water clarity is more consistently clear, of 0.5% yr^-1^, [28]. This may be an underrepresentation given that inshore meadows in turbid water are likely more vulnerable to both direct impact (dredging, land reclamation) and indirect impacts from sedimentation from land. This value is conservative compared to the global estimates of seagrass loss: 0.9% yr^-1^ pre-1940; and 7% yr^-1^ since 1990 [29].

**Rate of recovery following restoration.** It is assumed that there will be time lags following restoration before the desired ecosystem service is obtained. For land, we assumed it would take 10 years following restoration of riparian habitats before sediment erosion, the objective of the restoration projects, would be fully mitigated. There is considerable uncertainty in this parameter, with sediment loads taking variable lengths of time to decline following restoration, and in some instances increasing following restoration [30, 31]. The aim of marine restoration was to achieve high density plant coverage which was assumed to take 3 years [32].

**Marine habitat expansion rate.** The rate of expansion of seagrass habitats was 1.13% yr^-1^ based on the observed rate of expansion of seagrass in Tampa Bay, Florida, over 22 years, achieved by reductions in nitrogen from land [33]. Tampa Bay has a similar climate and size to Moreton Bay, and we expect that seagrass in Moreton Bay could respond similarly to reductions in land based impacts.

There is considerable uncertainty in this parameter. Seagrass growth rate can be quantified in several different ways. At the individual level, these metrics include shoot or leaf elongation, and rhizome horizontal and vertical elongation. Recruitment rate (individuals area^-1^ time^-1^) or productivity (dry weight area ^-1^ time^-1^) can provide estimates of seagrass growth at the population level. In this study, we were interested in the growth (and recovery) of seagrass meadows, typically quantified as a linear extension of an existing meadow or reduction in the size of gaps. We reviewed the literature for estimates of areal expansion of seagrass beds, which we standardized to the initial size of the meadow (% yr^-1^). We only included studies (n=13) that or provided calculations or values for measured areal extent of seagrass beds at two different times; this allowed us to calculate % change per unit time (**S2_Table**). These studies were mostly on single species. Most values were obtained from long-term monitoring of seagrass beds, except for a single study on restoration experiments using 3 seagrass species in Florida and Maryland USA. The median value for expansion rate of seagrass from data reported in the literature, mostly from very small scale studies, was 14% yr^-1^.

**Ecosystem service values.** The value of ecosystem services delivered by seagrass ($40,439 ha^-1^ yr^-1^) and riparian habitats ($41,665) was derived from [34] by adjusting values from 2007 USD to 2015 AUD. The value for seagrass is based on the value reported for seagrass/algae beds, and the value for riparian is based on the value reported for Swamps/Floodplains.

**Additional Sensitivity analyses**

Sensitivity analyses were conducted to examine the effect of varying some of the key model parameters. These included: The rates of seagrass decline and expansion, the rate and magnitude of land clearing, the functional form of the relationship between sediment loads and seagrass suitable habitat area, the maximum area of seagrass in a restoring state, the rate of revegetation of seagrass and riparian habitats, and the cost of restoration in seagrass and riparian habitats. Given the large number of potential parameter combinations we ran simulations using combinations of parameters that we thought would be most relevant to model interpretation. See **S3_Table** for further information.

The objective of our modelling was to maximise the extent of seagrass at the end of the project duration. We ran an additional analysis to examine how our decision making may vary if instead we aimed to maximise the value of ecosystem services of aquatic habitats comprised of both seagrass and riparian vegetation. For this analysis we calculated the increase in habitat area for both seagrass and riparian habitats at the end of the project duration, and then multiplied the area of each habitat by the value of ecosystem services delivered by that habitat (See **S1_Table** and “Ecosystem Service Values”, above). We then recorded which action returned the greatest total value of ecosystem services at the end of the project. Our calculations do not factor in inflation or discount rates, and are meant only to illustrate how aiming to maximize cost-effectiveness for the land and ocean ecosystems combined may change our decision making.

References

1. Argent RM, Perraud J-M, Rahman JM, Grayson RB, Podger GM. A new approach to water quality modelling and environmental decision support systems. Environmental Modelling and Software. 2009;24:809–18.

2. Chiew F, Peel M, Western A. Application and testing of the simple rainfall-runoff model SIMHYD. In: Singh VP, Frevert DK, editors. Mathematical models of small watershed hydrology and Applications. Colorado: Water Resources Publication; 2002. p. 335-67.

3. Jeffrey SJ, Carter JO, Moodie KB, Beswick AR. Using spatial interpolation to construct a comprehensive archive of Australian climate data. Environmental Modelling & Software. 2001;16(4):309-30. doi: <http://dx.doi.org/10.1016/S1364-8152(01)00008-1>.

4. DSITIA. Land use Summary: South East Queensland NRM Region. Department of Science, Information Technology, Innovation and the Arts, Queensland Government., 2014.

5. Anon. Queensland waterways for waterway barrier works. In: Agriculture and Fisheries QG, editor. Brisbane: Department of Agriculture and Fisheries; 2016.

6. Beaman RJ. 3D-GBR: A high-resolution depth model for the Great Barrier Reef and Coral Sea. Cairns, Australia: Reef and Rainforest Research Centre, 2010.

7. Saunders MI, Leon J, Phinn SR, Callaghan DP, O'Brien KR, Roelfsema CM, et al. Coastal retreat and improved water quality mitigate losses of seagrass from sea level rise. Glob Change Biol. 2013;19(8):2569-83. doi: 10.1111/gcb.12218.

8. Jerlov NG. Light: general introduction. In: Kinne O, editor. Marine Ecology, Environmental Factors. London: Wiley Interscience; 1970. p. 95-102.

9. Poole HH, Atkins WR. Photo-electric measurements of submarine illumination throughout the year. Journal of the Marine Biological Association of the U K. 1929;16:297-394.

10. Roelfsema CM, Phinn SR, Udy N, Maxwell P. An integrated field and remote sensing approach for mapping seagrass cover, Moreton Bay, Australia Spatial Science. 2009;54(1):45-62.

11. Callaghan D, Leon J, Saunders M. Results of wave modelling for Moreton Bay, Southeast Queensland, and a reef lagoon off Lizard Island, Great Barrier Reef, Australia. Supplement to: Callaghan, DP; Leon, JX; Saunders, MI (2015): Wave modelling as a proxy for seagrass ecological modelling: Comparing fetch and process-based predictions for a bay and reef lagoon. Estuarine, Coastal and Shelf Science, 153, 108-120, doi:10.1016/j.ecss.2014.12.016. 2015.

12. Callaghan DP, Leon JX, Saunders MI. Wave modelling as a proxy for seagrass ecological modelling: Comparing fetch and process-based predictions for a bay and reef lagoon. Estuarine, Coastal and Shelf Science. 2015;153(0):108-20. doi: <http://dx.doi.org/10.1016/j.ecss.2014.12.016>.

13. Venables WN, Ripley BD. Modern Applied Statistics with S. Fourth edition ed: Springer; 2002.

14. Olley J, Wilkinson S, Caitcheon G, Read A. Protecting Moreton Bay: How can we reduce sediment and nutrients loads by 50%? Proc 9th International River Symposium; Brisbane, Queensland, Australia2005. p. 1-9.

15. Olley J, Burton J, Hermoso V, Smolders K, McMahon J, Thomson B, et al. Remnant riparian vegetation, sediment and nutrient loads, and river rehabilitation in subtropical Australia. Hydrological Processes. 2015;29(10):2290-300. doi: 10.1002/hyp.10369.

16. Coates-Marnane J, Olley J, Burton J, Sharma A. Catchment clearing accelerates the infilling of a shallow subtropical bay in east coast Australia. Estuarine, Coastal and Shelf Science. 2016;174:27-40. doi: <http://dx.doi.org/10.1016/j.ecss.2016.03.006>.

17. Olds AD, Pitt KA, Maxwell PS, Babcock RC, Rissik D, Connolly RM. Marine reserves help coastal ecosystems cope with extreme weather. Glob Change Biol. 2014;20(10):3050-8. doi: 10.1111/gcb.12606.

18. Maggini R, Kujala H, Taylor M, Lee J, Possingham H, Wintle B, et al. Protecting and restoring habitat to help Australia’s threatened species adapt to climate change Gold Coast: National Climate Change Adaptation Research Facility, 2013.

19. Binney J, James D. Sharing the load: A collaborative approach to investing in South East Queensland's waterways. Brisbane QLD: Mainstream Economics and Policy, 2011.

20. Bayraktarov E, Saunders MI, Abdullah S, Mills M, Beher J, Possingham HP, et al. The cost and feasibility of marine coastal restoration. Ecol Appl. 2016;26(4):1055-74. doi: 10.1890/15-1077.

21. Anon. Review of mooring infrastruction technology Q0294 GCWA – Buoy Mooring Review Bundall Qld Australia: RPS Apasa Pty. Ltd., 2014.

22. DSITIA QDoS, Information Technology and Innovation. Land cover change in Queensland 2012–13 and 2013–14: a Statewide landcover and trees study (SLATS) report. DSITI, Brisbane: 2015.

23. DSITIA QDoS, Information Technology and Innovation. Land cover change in Queensland 2008–2009: a Statewide Landcover and Trees Study (SLATS) report. DSITI, Brisbane: 2010.

24. DSITIA QDoS, Information Technology and Innovation. Land cover change in Queensland 2009–2010: a Statewide Landcover and Trees Study (SLATS) report. DSITI, Brisbane: 2012.

25. DSITIA QDoS, Information Technology and Innovation. Land cover change in Queensland 2010–2011: a Statewide Landcover and Trees Study (SLATS) report. DSITI, Brisbane: 2014.

26. DSITIA QDoS, Information Technology and Innovation. Land cover change in Queensland 2011–2012: a Statewide Landcover and Trees Study (SLATS) report. DSITI, Brisbane: 2014.

27. Roelfsema C, Kovacs E, Saunders M, Phinn S, Lyons M, Maxwell P. Challenges of remote sensing for quantifying changes in large complex seagrass environments. Estuarine, Coastal and Shelf Science. 2013;133:161–71.

28. Lyons MB, Roelfsema CM, Phinn SR. Towards understanding temporal and spatial dynamics of seagrass landscapes using time-series remote sensing. Estuarine, Coastal and Shelf Science. 2013;120:42-53. doi: <http://dx.doi.org/10.1016/j.ecss.2013.01.015>.

29. Waycott M, Duarte CM, Carruthers TJB, Orth RJ, Dennison WC, Olyarnik S, et al. Accelerating loss of seagrasses across the globe threatens coastal ecosystems. Proceedings of the National Academy of Sciences. 2009;106(30):12377-81. doi: 10.1073/pnas.0905620106.

30. Collier KJ, Rutherford JC, Quinn JM, Davies-Colley RJ. Forecasting rehabilitation outcomes for degraded New Zealand pastoral streams. Water Science and Technology. 2001;43(9):175-84.

31. Meals DW, Dressing SA, Davenport TE. Lag time in water quality response to best management practices: A review. Journal of Environmental Quality. 2010;39(1):85-96. doi: 10.2134/jeq2009.0108.

32. Verduin J, Sinclair E. Seagrass meadow restoration trial using transplants – Cockburn Sound, Western Australia 2013 [26 May 2016]. Available from: <https://site.emrprojectsummaries.org/2013/03/08/seagrass-meadow-restoration-trial-using-transplants-cockburn-sound-western-australia/>.

33. Greening H, Janicki A. Toward reversal of eutrophic conditions in a subtropical estuary: water quality and seagrass response to nitrogen loading reductions in Tampa Bay, Florida, USA. Environmental Management. 2006;38(2):163-78. doi: 10.1007/s00267-005-0079-4.

34. Costanza R, de Groot R, Sutton P, van der Ploeg S, Anderson SJ, Kubiszewski I, et al. Changes in the global value of ecosystem services. Global Environmental Change. 2014;26:152-8. doi: <http://dx.doi.org/10.1016/j.gloenvcha.2014.04.002>.
