# Supplementary material for: Simple rules can guide whether land- or ocean-based conservation will best benefit marine ecosystems
Source: PLoS Biol. 2017 Sep 6;15(9):e2001886. doi: 10.1371/journal.pbio.2001886 (PMC5587113; doi:10.1371/journal.pbio.2001886)
Supplement: S1 Code — (DOCX) [file pbio.2001886.s022.docx]

**Supplemental Matlab Code For:** Simple rules can guide whether land or ocean based conservation will best benefit marine ecosystems

Megan I. Saunders^1,2,3,4,5*^, Michael Bode^1,2,6^, Scott Atkinson^1,2^, Carissa J. Klein^1,2,3^, Anna Metaxas^7^, Jutta Beher^1,2^, Maria Beger^1,2,8^, Morena Mills^1,2,9^, Sylvaine Giakoumi^1,2,10^, Vivitskaia Tulloch^1,2^, Hugh Possingham^1,2,11^

**Affiliations:**

1. Centre for Biodiversity and Conservation Science, The University of Queensland, St. Lucia, Australia 4072
2. Australian Research Council (ARC) Centre of Excellence in Environmental Decisions, University of Queensland, St Lucia, 4072 Australia
3. School of Earth and Environmental Sciences, The University of Queensland, St. Lucia, Australia, 4072
4. The Global Change Institute, The University of Queensland, St Lucia, Australia, 4072
5. School of Chemical Engineering, The University of Queensland, St. Lucia, Australia 4072
6. School of BioSciences, The University of Melbourne, Parkville, Victoria, Australia, 3010
7. Department of Oceanography, Dalhousie University, Halifax, Nova Scotia, Canada, B3H 4R2
8. School of Biology, University of Leeds, Leeds, LS2 9JT, UK
9. Department of Life Sciences, Imperial College London, Silwood Park Campus, Buckhurst Road, Ascot, Berkshire SL5 7PY, UK
10. Université Côte d’Azur, CNRS, FRE 3729 ECOMERS, Parc Valrose, 28 Avenue Valrose, 06108 Nice, France
11. The Nature Conservancy, Arlington VA, USA, 22203

* m.saunders1@uq.edu.au

%%% by Megan Saunders and Michael Bode

%%% Most recently updated in July 2017

function [ResultsVec,TV] = SystemDynamics_28_04_16(UV,B,T,PLOTTING)

% === INPUTS ===

% UV = a control vector indicating which action to invest in;

% B = Budget;

% T = project timeline;

% PLOTTING=On or off, 1 or 0

%% EXTRACT DATA FROM THE SPREADSHEET IN WHICH THE PARAMETERS ARE STORED

[dd,tt] = xlsread('Parameters_21042016.xlsx');

%% DEFINE THE PROJECT PARAMETERS

dt = dd(3,1); % discretisation of time in the model

T = dd(2,1); % Project duration

Time = dt:dt:T; % Project timeline

nact = 4; % Number of actions to simulate (+ do nothing scenario)

%% EXTRACT INITIAL CONDITIONS FROM THE SPREADSHEET

P_S_i = dd(4,1); % Sea - Protected Suitable

R_S_i = dd(5,1); % Sea - Restoring Suitable

A_S_i = dd(6,1); % Sea - Available Suitable

C_S_i = dd(7,1); % Sea - Cleared Suitable

U_S_i = dd(8,1); % Sea - Unsuitable

P_L_i = dd(9,1); % Land - Protected

R_L_i = dd(10,1); % Land - Restoring

A_L_i = dd(11,1); % Land - Available

C_L_i = dd(12,1); % Land – Cleared

% Define the max tonnage of suspended sediment which would be delivered to the ocean in the a year _IF_ all riparian areas are cleared

SS_max = dd(24,1);

% Calculate the total area of ocean habitat, suitable ocean habitat, and land

TotalSea = P_S_i + R_S_i + A_S_i + C_S_i + U_S_i;

SuitableSea_i = TotalSea - U_S_i;

TotalLand = P_L_i + R_L_i + A_L_i + C_L_i;

% Normalise the land and sea components so that they add up to 1 (i.e., express them as proportions)

% The proportion each category in the ocean which is suitable for seagrass

P_S_p = P_S_i / SuitableSea_i;

R_S_p = R_S_i / SuitableSea_i;

A_S_p = A_S_i / SuitableSea_i;

C_S_p = C_S_i / SuitableSea_i;

%The proportion of land in each category

P_L_p = P_L_i / TotalLand;

R_L_p = R_L_i / TotalLand;

A_L_p = A_L_i / TotalLand;

C_L_p = C_L_i / TotalLand;

%% EXTRACT THE SYSTEM PARAMETERS FROM THE EXCEL SPREADSHEET

F_SR = dd(13,1)./100; % Feasibility of restoring seagrass

F_LR = dd(14,1)./100; % Feasibility of restoring riparian areas

g_S = dd(15,1)./100; % Growth rate of seagrass

d_S = dd(16,1)./100; % Loss rate seagrass in UNPROTECTED areas

d_LL = dd(17,1)./100; % Rate of loss of available land

g_SR = dd(18,1)./100; % Rate of revegetation of seagrass

g_LL = dd(19,1)./100; % Rate of revegetation on the land

C_SR = dd(20,1).*SuitableSea_i; % Cost of seagrass restoration

C_SP = dd(21,1).*SuitableSea_i; % Cost of seagrass protection

C_LR = dd(22,1).*TotalLand; % Cost of land restoration

C_LP = dd(23,1).*TotalLand; % Cost of land protection

PS_max = dd(25,1)./100; % Percent of seagrass habitat which can be protected

RS_max = dd(26,1)./100; % Percent of seagrass habitat which can be restored

RL_max = dd(27,1)./100; % Percent of riparian habitat which can be restored

%% DESCRIBE HOW MUCH MONEY WE WILL ALLOCATE TO EACH ACTION

if nargin == 0

% {'P-S';'R-PS';'R-US';'P-RH';'R-RH';'DN'}; % Names for the actions

% If we don't put any inputs into the function, use the following defaults

% U is the series of U_SP... etc

U_SP = 1; % Allocation to seagrass protection

U_SR = 0; % Allocation to seagrass restoration

U_LP = 0; % Allocation to protection on the land

U_LR = 0; % Allocation to restoration on the land

B = dd(1,1); % Total annual budget in $/year

PLOTTING = 1; % if 0 there is no plotting later, if 1 there is

F_ax = 1; % in the title of the plot gives the dominant action

else

% If we do put inputs into the function using the second set of code,

U_SP = UV(1); U_SR = UV(2); U_LP = UV(3); U_LR = UV(4);

[~,F_ax] = max(UV);

end

%% run the following loop which updates the values based on the background rates of change and the conservation actions through time

%% Preallocate variables

ntime = length(Time);

P_S_L = zeros(1,ntime);

R_S_L = zeros(1,ntime);

A_S_L = zeros(1,ntime);

C_S_L = zeros(1,ntime);

U_S_L = zeros(1,ntime);

P_L_L = zeros(1,ntime);

R_L_L = zeros(1,ntime);

A_L_L = zeros(1,ntime);

C_L_L = zeros(1,ntime);

% vectors for the sea component that will update each loop based on sediment input.

% Used later to update the vectors above

P_S_L_link = zeros(1,ntime);

R_S_L_link = zeros(1,ntime);

A_S_L_link = zeros(1,ntime);

C_S_L_link = zeros(1,ntime);

% vectors for land similar to sea vectors - only so that we stay consistent later in loop

P_L_L_link = zeros(1,ntime);

R_L_L_link = zeros(1,ntime);

A_L_L_link = zeros(1,ntime);

C_L_L_link = zeros(1,ntime);

% Preallocate vectors for sediment, suitable substrate, and the amount of habitat lost

% or gained by changes in sediment

Sediment = zeros(1,ntime); % sediment run-off in tonnes

Suitable = zeros(1,ntime); % area of suitable substrate in Ha

xs_Suitable = zeros(1,ntime); % area of additional substrate in Ha

xs_Unsuitable = zeros(1,ntime); % area of lost substrate in Ha

%% Simulate the system dynamics through time

for t = 1:ntime % Store the proportion values in vectors

if t==1

% set the values in each vector for the first timestep.

P_S_L(t) = P_S_p;

R_S_L(t) = R_S_p;

A_S_L(t) = A_S_p;

C_S_L(t) = C_S_p;

U_S_L(t) = U_S_i; % NB: This variable has units of hectares, rather than percentages

P_L_L(t) = P_L_p;

R_L_L(t) = R_L_p;

A_L_L(t) = A_L_p;

C_L_L(t) = C_L_p;

P_S_L_link(t) = P_S_p;

R_S_L_link(t) = R_S_p;

A_S_L_link(t) = A_S_p;

C_S_L_link(t) = C_S_p;

P_L_L_link(t) = P_L_p;

R_L_L_link(t) = R_L_p;

A_L_L_link(t) = A_L_p;

C_L_L_link(t) = C_L_p;

% How much sediment going to ocean in each timestep according to land uses?

Sediment(t) = SS_max*(1 - (A_L_p + P_L_p));

Suitable(t) = SuitableSea_i; % Vector of habitat area (in hectares) that is suitable

xs_Suitable(t) = 0; % in Ha

xs_Unsuitable(t) = 0; % in Ha

end

if t > 1

% How much sediment going into bay based on intact land?

Sediment(t) = SS_max.*(1 - (A_L_L(t-1) + P_L_L(t-1)));

% How much suitable seagrass habitat is there, given this sediment load?

Suitable(t) = max(0,(23567.71 - 5.643e-9.*Sediment(t).^2));

xs_Suitable(t) = Suitable(t) - Suitable(t-1); % in Ha

xs_Unsuitable(t) = -1*(xs_Suitable(t)); % in ha

U_S_L(t) = TotalSea - Suitable(t); % in Ha

%% These vectors store the values for the new habitat calculations

%% areas based on the sediment run-off in this time-step

if xs_Suitable(t) > 0 % if sediment has decreased and water quality increased, creating more suitable habitat

P_S_L_link(t) = P_S_L(t-1)*Suitable(t-1)./Suitable(t); %get protected in Ha and then divide by new suitable to get new proportion protected

R_S_L_link(t) = R_S_L(t-1)*Suitable(t-1)./Suitable(t);

A_S_L_link(t) = A_S_L(t-1)*Suitable(t-1)./Suitable(t);

C_S_L_link(t) = (C_S_L(t-1)*Suitable(t-1) + xs_Suitable(t))./Suitable(t); % add the new habitat to the Cleared fraction, and let seagrass grow into it in the time step

P_L_L_link(t) = P_L_L(t-1);

R_L_L_link(t) = R_L_L(t-1);

A_L_L_link(t) = A_L_L(t-1);

C_L_L_link(t) = C_L_L(t-1);

else % if sediment has increased and water quality decreased, create less suitable habitat

P_S_L_link(t) = P_S_L(t-1);

R_S_L_link(t) = R_S_L(t-1);

A_S_L_link(t) = A_S_L(t-1);

C_S_L_link(t) = C_S_L(t-1);

P_L_L_link(t) = P_L_L(t-1);

R_L_L_link(t) = R_L_L(t-1);

A_L_L_link(t) = A_L_L(t-1);

C_L_L_link(t) = C_L_L(t-1);

end

%%%% Calculate the amount of habitat converted in each time step

%% Sea

%% Marine Restoration

if R_S_L_link(t) < RS_max

RestoreS = min(dt*F_SR*U_SR*B/C_SR,C_S_L_link(t)); % Restoration work in the sea (C_S to R_S)

else

RestoreS = 0;

end

%% Marine Revegetation

RevegS = dt*g_SR*R_S_L_link(t); % Rate of revegetation following restoration (R_S to P_S)

%% Marine Protection

if sum(P_S_L_link) <= PS_max

ProtectS = min(dt*U_SP*B/C_SP,A_S_L_link(t)); % Protection work in the sea (A_S to P_S)

else

ProtectS = 0;

end

%% Marine Degradation

ClearS = dt*d_S*A_S_L_link(t); % Clearance of available habitat in the sea (A_S to C_S)

%% Marine Regeneration

GrowthS = min(dt*g_S*(A_S_L_link(t)+P_S_L_link(t)),C_S_L_link(t)); % Growth of seagrass into newly available habitat (C_S to A_S)

%%%% Land

% limit the amount of the catchment available for restoration

if P_L_L(t) + R_L_L(t) < RL_max

% Land restoration

RestoreL = min(dt*F_LR*U_LR*B/C_LR,C_L_L_link(t)); % C to R

else

RestoreL = 0;

end

%% Land Revegtation

RevegRL = dt*g_LL*R_L_L_link(t); % R to P

%% Land Protection

ProtectL = min(dt*U_LP*B/C_LP,A_L_L_link(t)); % A to P

%% Land Clearing

ClearL = dt*d_LL*A_L_L_link(t); % A to C

%%%%%%% update each parameter based on the values calculated previously

% Sea

R_S_L(t) = R_S_L_link(t) + RestoreS - RevegS;

C_S_L(t) = C_S_L_link(t) - RestoreS + ClearS - GrowthS;

P_S_L(t) = P_S_L_link(t) + RevegS + ProtectS;

A_S_L(t) = A_S_L_link(t) - ProtectS - ClearS + GrowthS;

% Land

R_L_L(t) = R_L_L_link(t) + RestoreL - RevegRL;

C_L_L(t) = C_L_L_link(t) - RestoreL + ClearL;

P_L_L(t) = P_L_L_link(t) + RevegRL + ProtectL;

A_L_L(t) = A_L_L_link(t) - ProtectL - ClearL;

end

end

%% PLOTTING

TV = dt:dt:T;

AX = {'P-S';'R-S';'P-RH';'R-RH';'DN'}; % Labels for axes

if PLOTTING == 1

figure(1), clf, TA = tight_subplot(1,2,0.075,0.075,0.075);

StackedBar_Sea = cumsum([P_S_L; R_S_L; A_S_L; C_S_L]);

StackedBar_Land = cumsum([P_L_L; R_L_L; A_L_L; C_L_L]);

COLORS = parula(11); COLORS = COLORS([1,3,5,7,9,11,2,4,6,8],:);

% Plot the state of the landscape through time

axes(TA(1)), hold on, box on

for i = 1:4

Br=bar(TV,100*StackedBar_Land(5-i,:),1);

set(Br,'edgecolor','none','facecolor',COLORS(i+5,:));

end,

axis tight,

% L = legend('Cleared land','Available land','Restoring land','Protected land',-1); set(L,'fontsize',16)

% turn this on if you want the legend

ylabel('Landscape dynamics (% of habitat)','fontsize',18);

xlabel('Project timeline (years)','fontsize',18);

title(['Action = ' AX{F_ax}],'fontsize',18);

% after "axis tight"

% Plot the state of the seascape through time

axes(TA(2)), hold on, box on

for i = 1:4

Br=bar(TV,100*StackedBar_Sea(5-i,:),1);

set(Br,'edgecolor','none','facecolor',COLORS(i,:));

end,

axis tight,

%L = legend('Cleared land','Available land','Restoring land','Protected land',-1); set(L,'fontsize',16);

ylabel('Seascape dynamics (% of habitat)','fontsize',18);

xlabel('Project timeline (years)','fontsize',18);

title(['Budget = ' num2str(B)],'fontsize',18);

% Output the figures as TIFFs

set(gcf, 'paperunits', 'centimeters', 'paperposition', [0 0 30 14]*0.8)

eval(['print -dtiff -r100 ../Matlab/Figures/Action_' AX{F_ax} '.tiff'])

end

%%% ResultsVec stores the results which are then used in the next function

ResultsVec = [P_S_L; R_S_L; A_S_L; C_S_L; U_S_L; P_L_L; R_L_L; A_L_L; C_L_L; Suitable; Sediment];

**Second file: invests the budget into each action independently**

clear all

close all

%% Import parameters from the excel spreadsheet

[dd,tt] = xlsread('Parameters_21042016.xlsx');

dt = dd(3,1); % discretisation of time in the model

T = dd(2,1);

Time = dt:dt:T;

ntime = length(Time);

nact= 4 % number of actions to take (4 actions plus do nothing)

% Get initial conditions for marine habitats from spreadsheet and calculate

% area of sea

P_S_i = dd(4,1); %Sea - protected suitable

R_S_i = dd(5,1); %Sea - restoring suitable

A_S_i = dd(6,1); %Sea - available suitable

C_S_i = dd(7,1); %Sea - cleared suitable

U_S_i = dd(8,1); %Sea - unsuitable

% calculate area of sea. This is used later to convert the proportion data

% back to Hectares

TotalSea = P_S_i + R_S_i + A_S_i + C_S_i + U_S_i;

%%

% UV(1) = 0; % U_SP;

% UV(3) = 0; % U_SR;

% UV(4) = 0; % U_LP;

% UV(5) = 1; % U_LR;

COLS = parula(nact+1);

figure(1), clf, hold on

for i = 1:nact+1 %go through the code in the system dynamics file for 5 loops. In loops 1-4 invest all the budget in one of the 4 actions. in loop 5 invest nothing for the do nothing action

T = dd(2,1); % Project time length

if i <= nact % First go through all the individual actions

B = dd(1,1); % Total annual budget;

UV = zeros(1,nact);

UV(i) = 1;

else % Then, go through the "do nothing" (no investment) action

B = 0;

UV = zeros(1,nact);

UV(5) = 0.00000000000000000000000000000000000000000001; %this is a very small value - kept here so that the plot title in “systems dynamics” indexes to "Do nothing" instead or rewriting over the P-S Plot

end

[ResultsVec,T] = SystemDynamics_28_04_16(UV,B,T,1);

% get the amount of each variable though time

P_S = ResultsVec(1,:); % Protected seagrass

R_S = ResultsVec(2,:); % Restoring seagrass

A_S = ResultsVec(3,:); % Available seagrass

C_S = ResultsVec(4,:); % Cleared seagrass

U_S = ResultsVec(5,:); % Unsuitable seagrass in Ha

P_L = ResultsVec(6,:); % Protected land

R_L = ResultsVec(7,:); % Restoring land

A_L = ResultsVec(8,:); % Available land

C_L = ResultsVec(9,:); % Cleared land

Suitable = ResultsVec(10,:); % Ha of suitable habitat in each time step

Sediment = ResultsVec(11,:); % Sediment tonnes in each time step

% save the amount of each variable at the end of the time frame for each

% action. Rows = different variables. Columns = Different actions

LogR(1,i) = P_S(end);

LogR(2,i) = R_S(end);

LogR(3,i) = A_S(end);

LogR(4,i) = C_S(end);

LogR(5,i) = U_S(end);

LogR(6,i) = P_L(end);

LogR(7,i) = R_L(end);

LogR(8,i) = A_L(end);

LogR(9,i) = C_L(end);

LogR(10,i) = Suitable(end);

LogR(11,i) = Sediment(end);

%Save the amount of each variable through time in a Cell array. Rows = variables;

%Columns = Different Actions; Cell contents = Variable for each action

%through time.

LogRc{1,i} = P_S;

LogRc{2,i} = R_S;

LogRc{3,i} = A_S;

LogRc{4,i} = C_S;

LogRc{5,i} = U_S;

LogRc{6,i} = P_L;

LogRc{7,i} = R_L;

LogRc{8,i} = A_L;

LogRc{9,i} = C_L;

LogRc{10,i} = Suitable;

LogRc{11,i} = Sediment;

SGintact_end(i) = (P_S(end) + A_S(end)).*Suitable(end);

end

%%%%% Make Plots %%%%

NME = {'P_S','R_S','A_S','C_S','P_L','R_L','A_L','C_L','Sediment'};

AX = {'P-S';'R-S';'P-RH';'R-RH';'DN'}

LabelSize = 14

%%%%%

%% Plot line plot of sediment though time for each action (lines)

figure(1), clf, hold on

for i=1:nact

P = plot(Time, LogRc{11,i,:}./LogRc{11,5,:}, 'linewidth', 13-i*2)

set(gca,'FontSize',20)

end

L = legend('Protect Sea', 'Restore Sea', 'Protect Land', 'Restore Land', 'location', 'westoutside');

ylabel('Sediment run-off')

xlabel('Time (years)')

%% Plot line plot of total seagrass suitable area though time for each action (lines)

figure(2), clf, hold on

for i=1:nact

P = plot(Time, LogRc{10,i,:}./LogRc{10,5,:}, 'linewidth', 13-i*2)

set(gca,'FontSize',20)

end

L = legend('Protect Sea', 'Restore Sea', 'Protect Land', 'Restore Land', 'location', 'westoutside');

ylabel('Seagrass suitable area')

xlabel('Time (years)')

%% Plot line plot of protected seagrass area though time for each action (lines)

figure(3), clf, hold on

for i=1:nact

P = plot(Time, (LogRc{1,i,:}.*LogRc{10,i,:})./(LogRc{1,5,:}.*LogRc{10,5,:}), 'linewidth', 13-i*2)

set(gca,'FontSize',20)

end

L = legend('Protect Sea', 'Restore Sea', 'Protect Land', 'Restore Land', 'location', 'westoutside');

ylabel('Protected seagrass area')

xlabel('Time (years)')

%% Plot line plot of unprotected seagrass area though time for each action (lines)

figure(4), clf, hold on

for i=1:nact

P = plot(Time, (LogRc{3,i,:}.*LogRc{10,i,:})./(LogRc{3,5,:}.*LogRc{10,5,:}), 'linewidth', 13-i*2)

set(gca,'FontSize',20)

end

L = legend('Protect Sea', 'Restore Sea', 'Protect Land', 'Restore Land', 'location', 'westoutside');

ylabel('Unprotected seagrass area')

xlabel('Time (years)')

%% Plot line plot of restoring seagrass area though time for each action (lines)

figure(5), clf, hold on

for i=1:nact

P = plot(Time, (LogRc{2,i,:}.*LogRc{10,i,:})./(LogRc{2,5,:}.*LogRc{10,5,:}), 'linewidth', 13-i*2)

set(gca,'FontSize',20)

end

L = legend('Protect Sea', 'Restore Sea', 'Protect Land', 'Restore Land', 'location', 'westoutside');

ylabel('Restoring seagrass area')

xlabel('Time (years)')

% Plot line plot of total seagrass area though time for each action (lines)

figure(6), clf, hold on

for i=1:nact

P = plot(Time, (LogRc{1,i,:}+LogRc{3,i,:}).*LogRc{10,i,:}./((LogRc{1,5,:}+LogRc{3,5,:}).*LogRc{10,5,:}), 'linewidth', 13-i*2)

set(gca,'FontSize',20)

end

L = legend('Protect Sea', 'Restore Sea', 'Protect Land', 'Restore Land', 'location', 'westoutside');

ylabel('Intact seagrass area')

xlabel('Time (years)')

%% Plot line plot of cleared seagrass area though time for each action (lines)

figure(7), clf, hold on

for i=1:nact

P = plot(Time, LogRc{4,i,:}.*LogRc{10,i,:}./(LogRc{4,5,:}.*LogRc{10,5,:}), 'linewidth', 13-i*2)

set(gca,'FontSize',20)

end

L = legend('Protect Sea', 'Restore Sea', 'Protect Land', 'Restore Land', 'location', 'westoutside');

ylabel('Cleared seagrass suitable area')

xlabel('Time (years)')

%% Print figures

% Output the results into a saved figure

set(figure(1), 'paperunits', 'centimeters', 'paperposition', [0 0 30 15])

print -dtiff -r150 ../Matlab/Figures/Sediment.tiff %% change file directories as required

set(figure(2), 'paperunits', 'centimeters', 'paperposition', [0 0 30 15])

print -dtiff -r150 ../Matlab/Figures/Potentialhabitat.tiff

set(figure(3), 'paperunits', 'centimeters', 'paperposition', [0 0 30 15])

print -dtiff -r150 ../Matlab/Figures/Protected.tiff

set(figure(4), 'paperunits', 'centimeters', 'paperposition', [0 0 30 15])

print -dtiff -r150 ../Matlab/Figures/Unprotected.tiff

set(figure(5), 'paperunits', 'centimeters', 'paperposition', [0 0 30 15])

print -dtiff -r150 ../Matlab/Figures/Restoring.tiff

set(figure(6), 'paperunits', 'centimeters', 'paperposition', [0 0 30 15])

print -dtiff -r150 ../Matlab/Figures/AllSeagrass.tiff

set(figure(7), 'paperunits', 'centimeters', 'paperposition', [0 0 30 15])

print -dtiff -r150 ../Matlab/Figures/Clearedsea.tiff
